# Supplementary figures and images for: Low cost and open source multi-fluorescence imaging system for teaching and research in biology and bioengineering
Source: PLoS One. 2017 Nov 15;12(11):e0187163. doi: 10.1371/journal.pone.0187163 (PMC5687719; doi:10.1371/journal.pone.0187163)

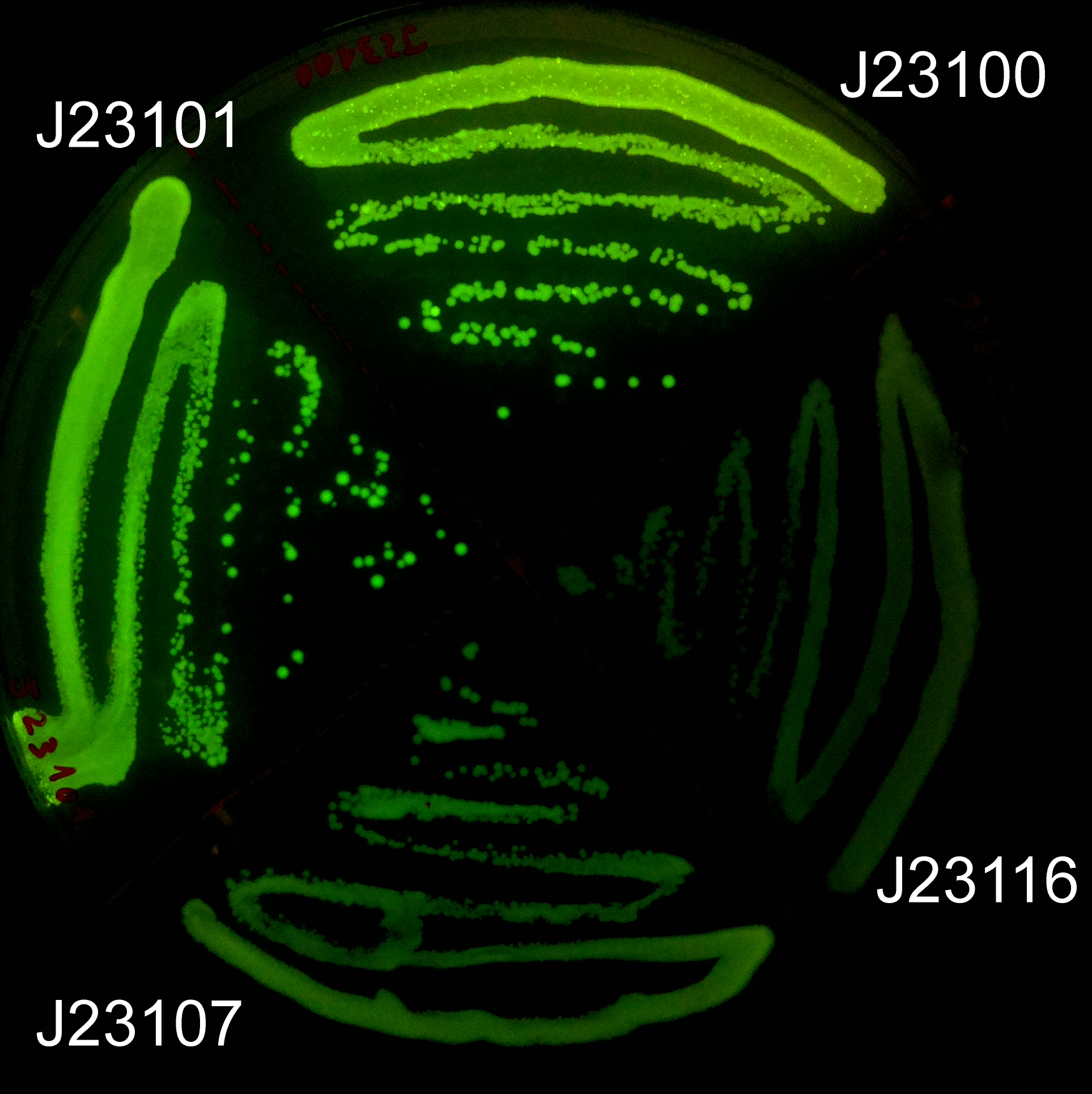

Supplement: S1 Fig — BCD12 RBS and B0015 terminator was used for all the combinations. (TIF) [file pone.0187163.s001.tif]

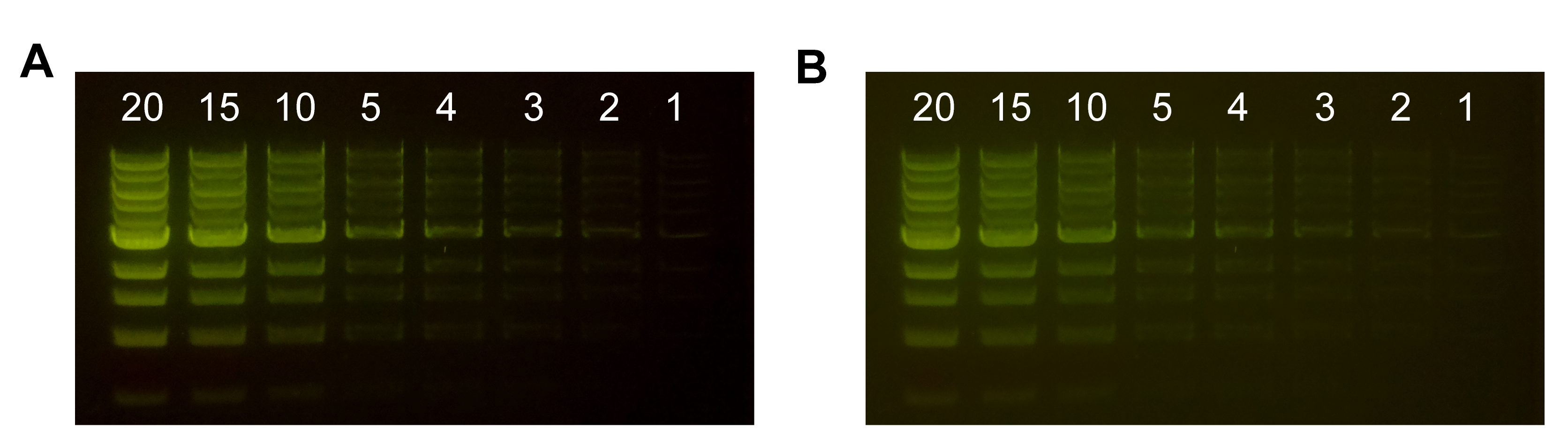

Supplement: S2 Fig — Gel loaded with 20, 15, 10, 5, 4,3,2 and 1 l of 1 Kb Ladder (NEB) labelled with SYBR-Safe. and imaged using raspistill command: -t 5000 -ss 240000 -ISO 300 -awbg 1,1 -co 30. (B) using raspistill command: -t 5000 -ss 170000 -ISO 300 -awbg 1,1 -co 0. (TIF) [file pone.0187163.s002.tif]

## PCB Components Mounting Details

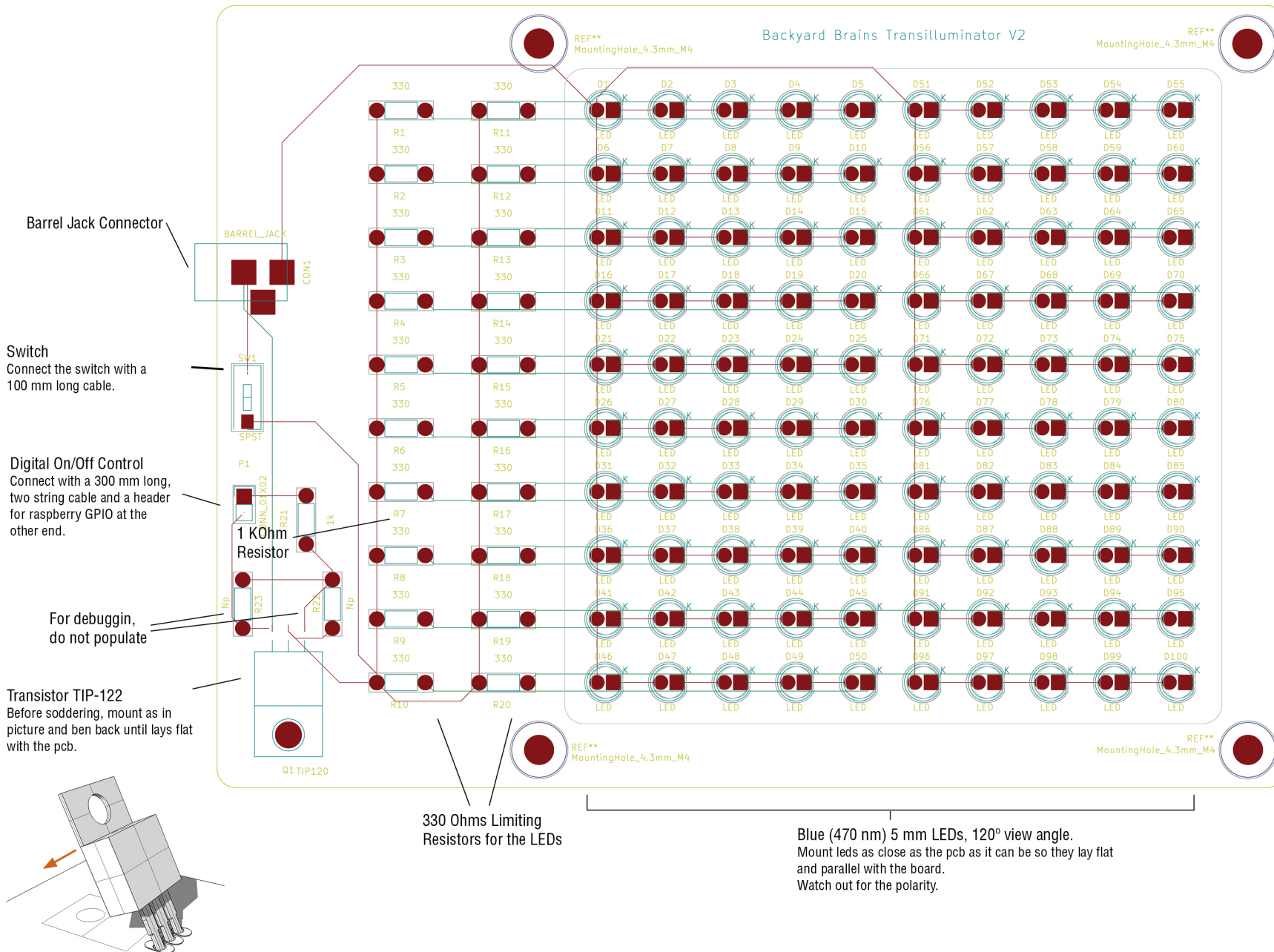

Supplement: S4 File — (PDF) [file pone.0187163.s006.pdf]
