# Supplementary material for: Low cost and open source multi-fluorescence imaging system for teaching and research in biology and bioengineering
Source: PLoS One. 2017 Nov 15;12(11):e0187163. doi: 10.1371/journal.pone.0187163 (PMC5687719; doi:10.1371/journal.pone.0187163)
Supplement: S1 File — (PDF) [file pone.0187163.s003.pdf]

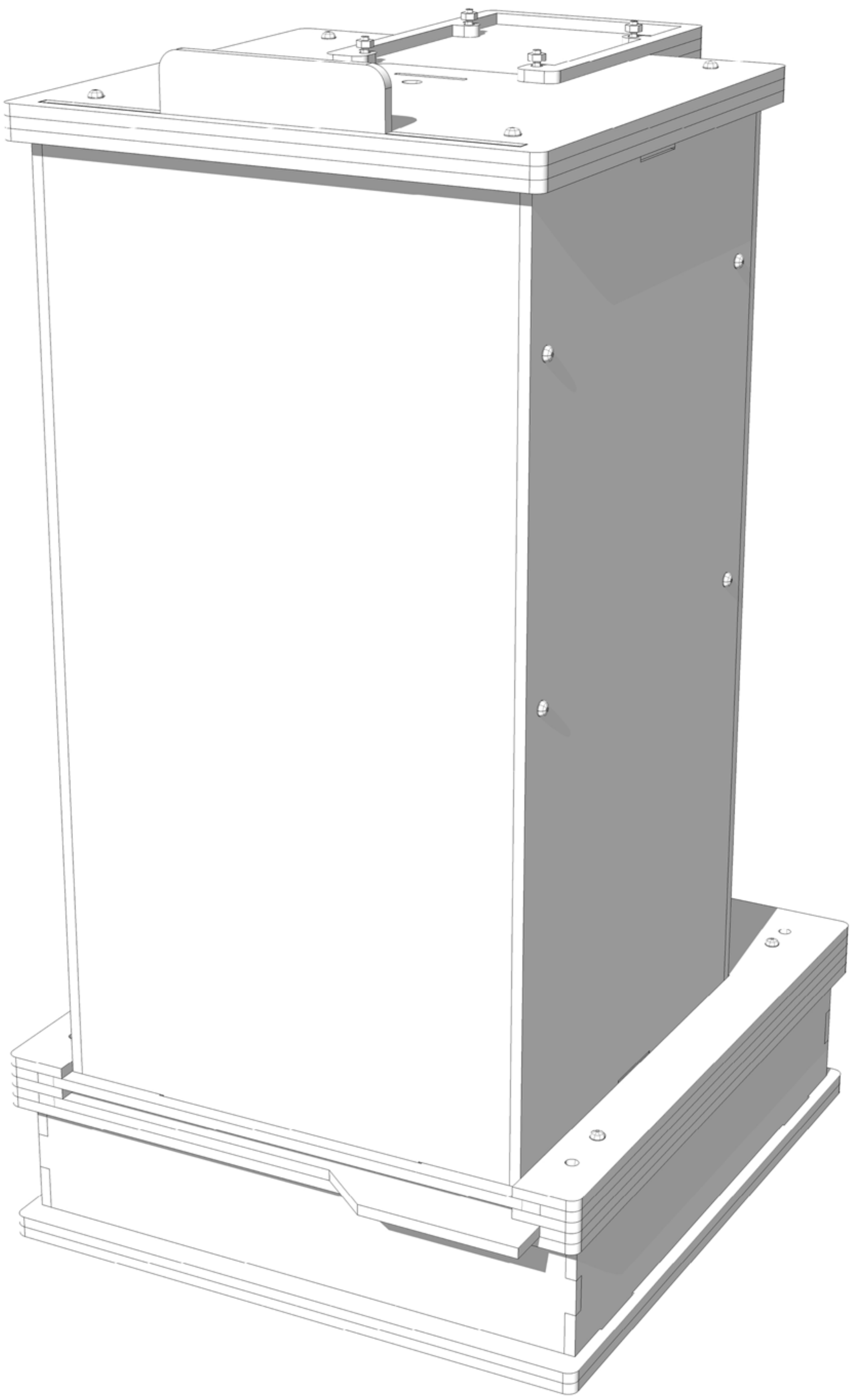

Fluorescence Imaging Station

Top Module Explosion

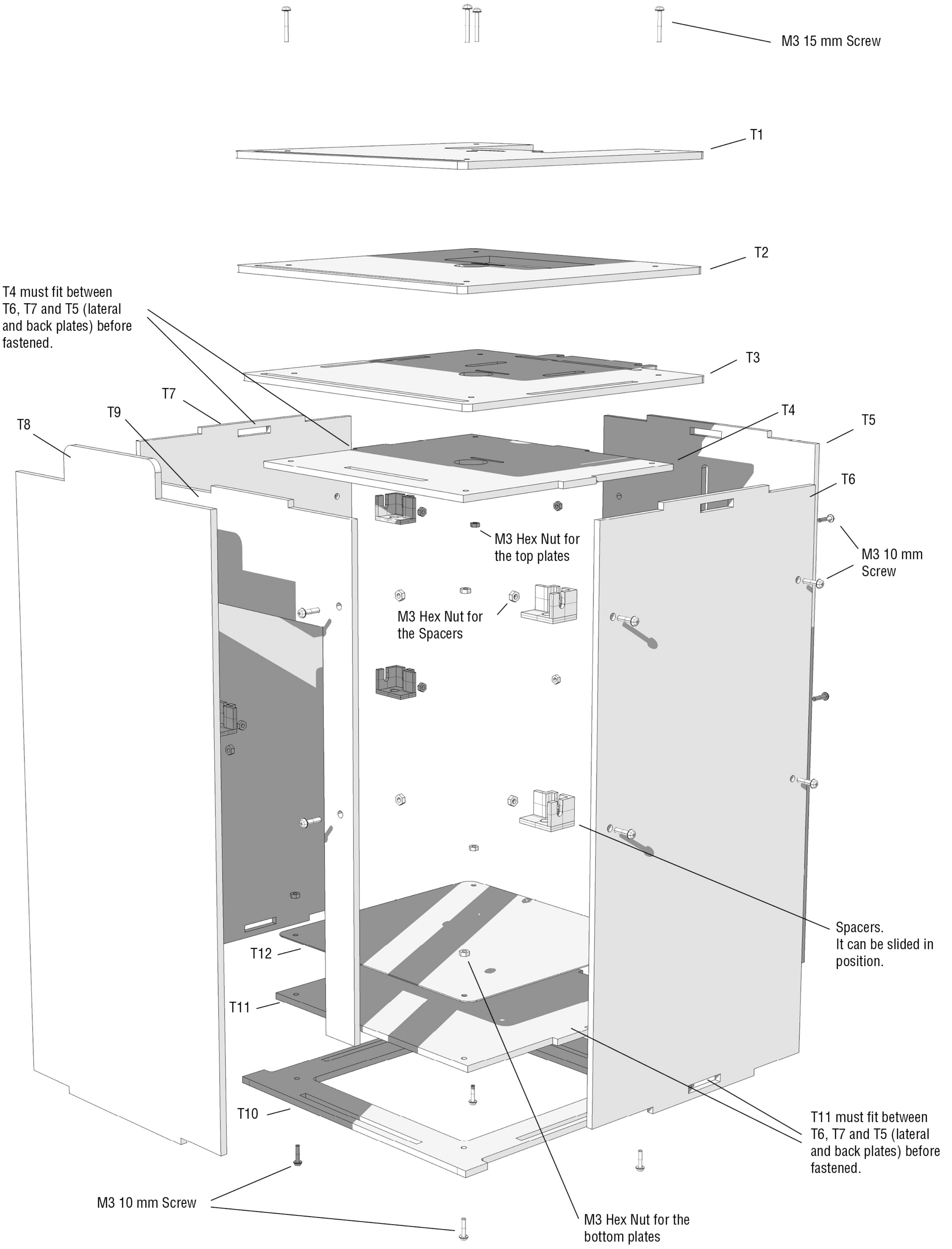

Top Module Parts

T1

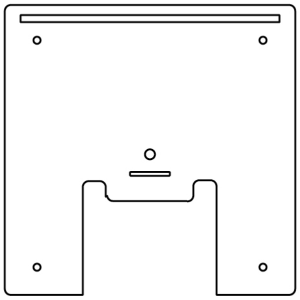

T2

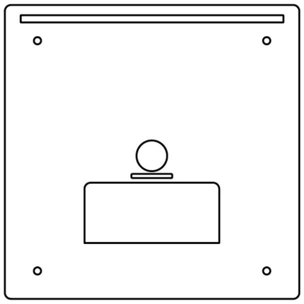

T3

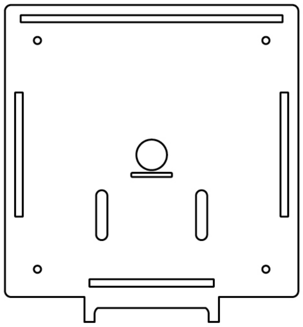

T4

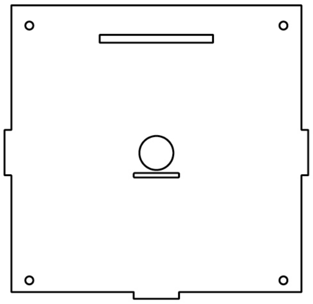

T5

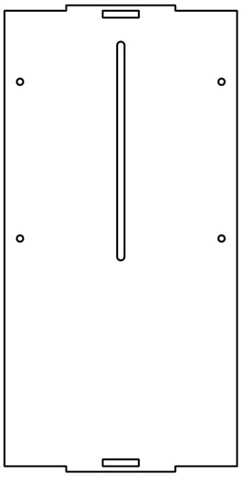

T6

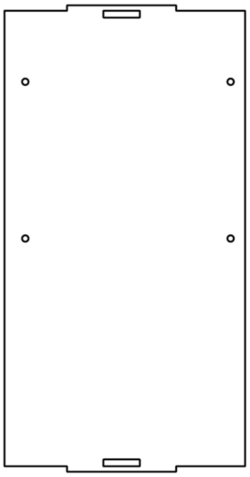

T7

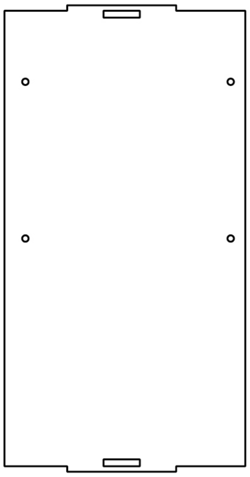

T8

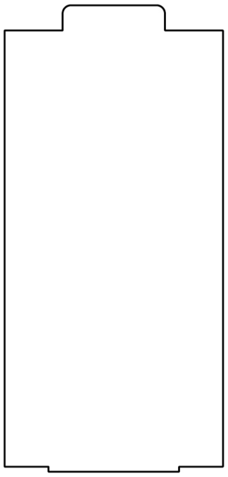

T9

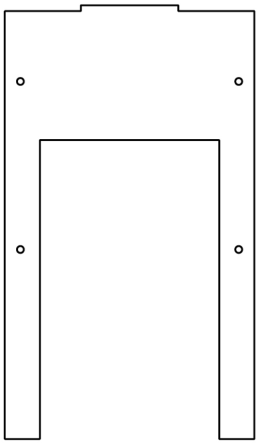

T10

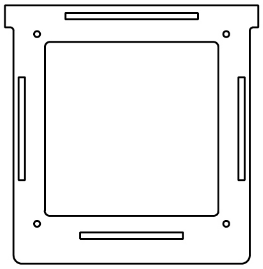

T11

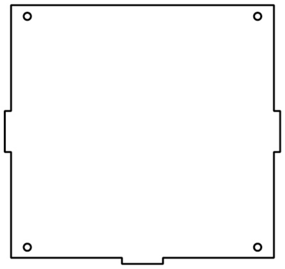

T12

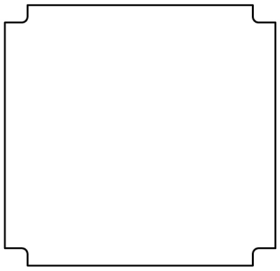

Screws and Nuts

4 M3 15 mm long screws and nuts for the top cover.

16 M3 10 mm long screws and nuts for the vertical plates.

4 M3 10 mm long screws and nuts for the botom cover.

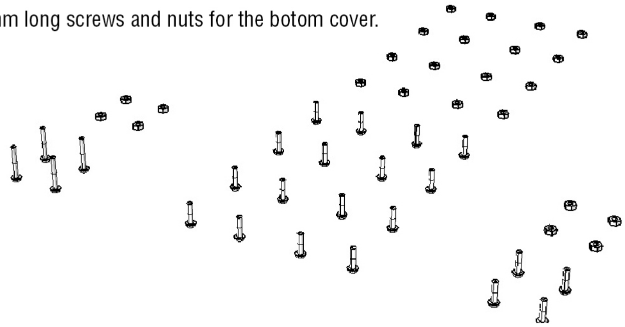

4 Spacers R and 4 Spacers L

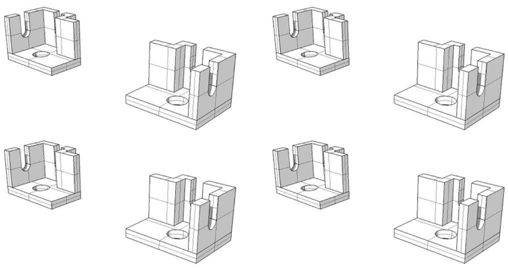

Top Module Assembly

Step 1

Place the M3 10 mm screws and nuts loosely in the holes on the vertical plates (T5, T6, T7, T9) as shown in the picture.

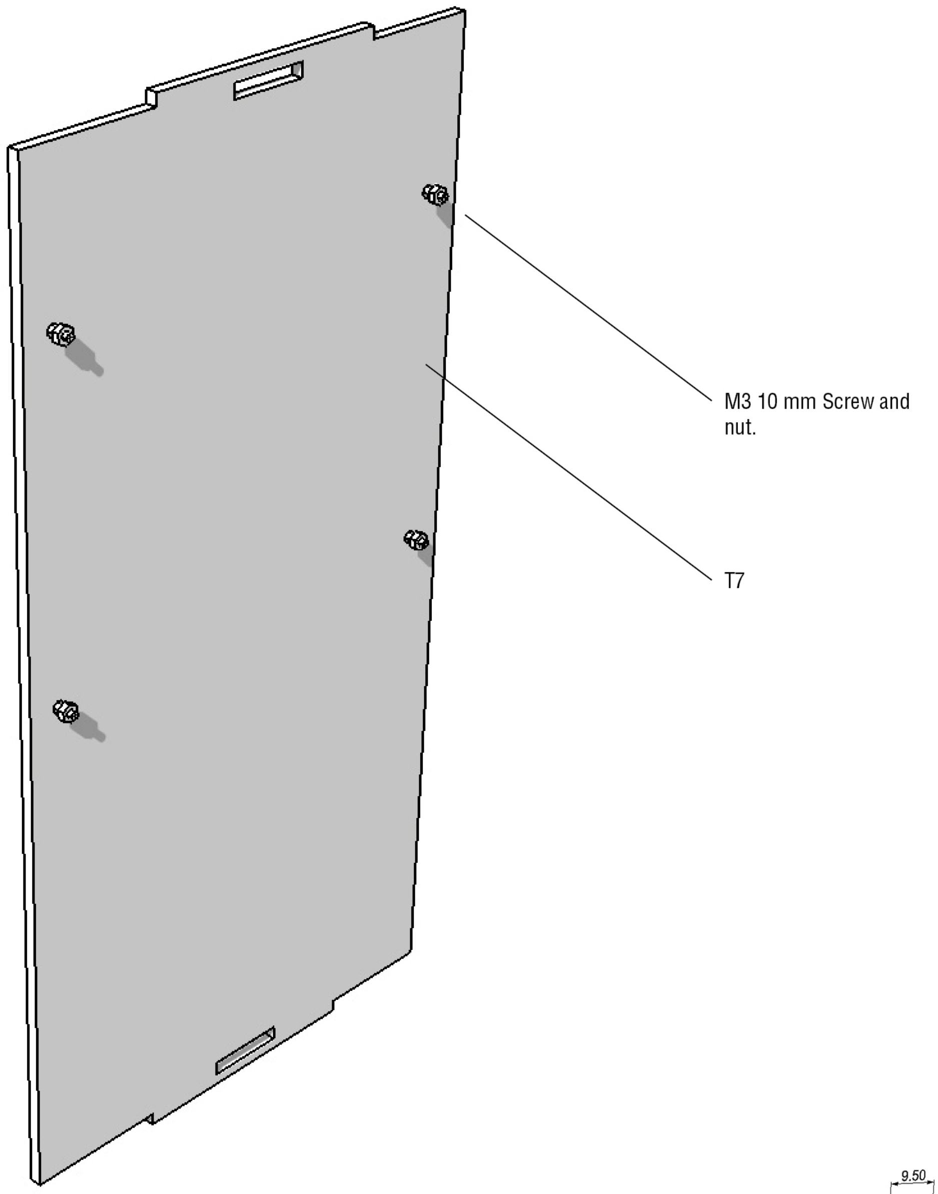

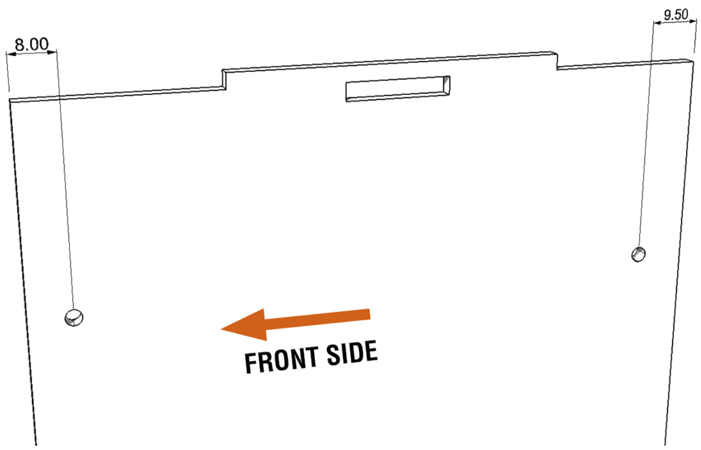

Be aware of the front side of the lateral plates (T6 and T7).

Step 2

Slide the spacers up until they fit completely and the tighten the screws to form the vertical structure of the top module.

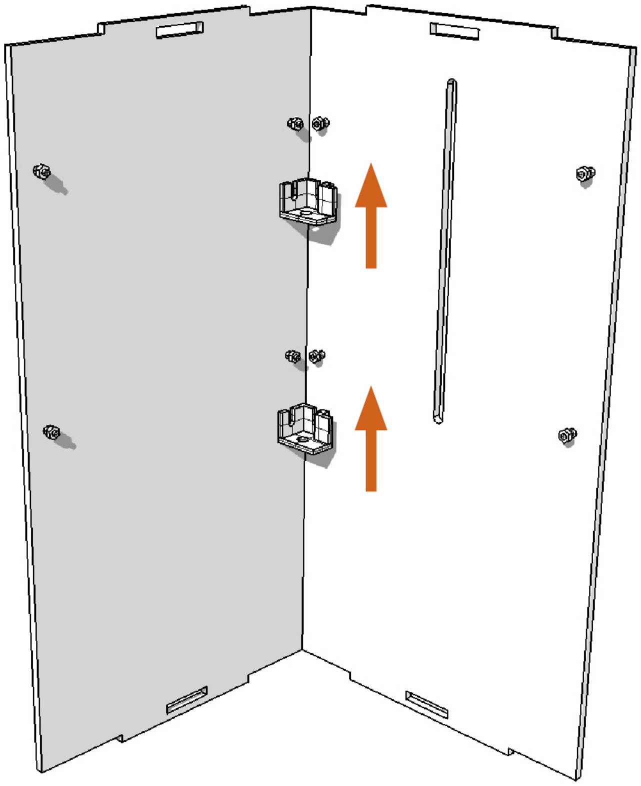

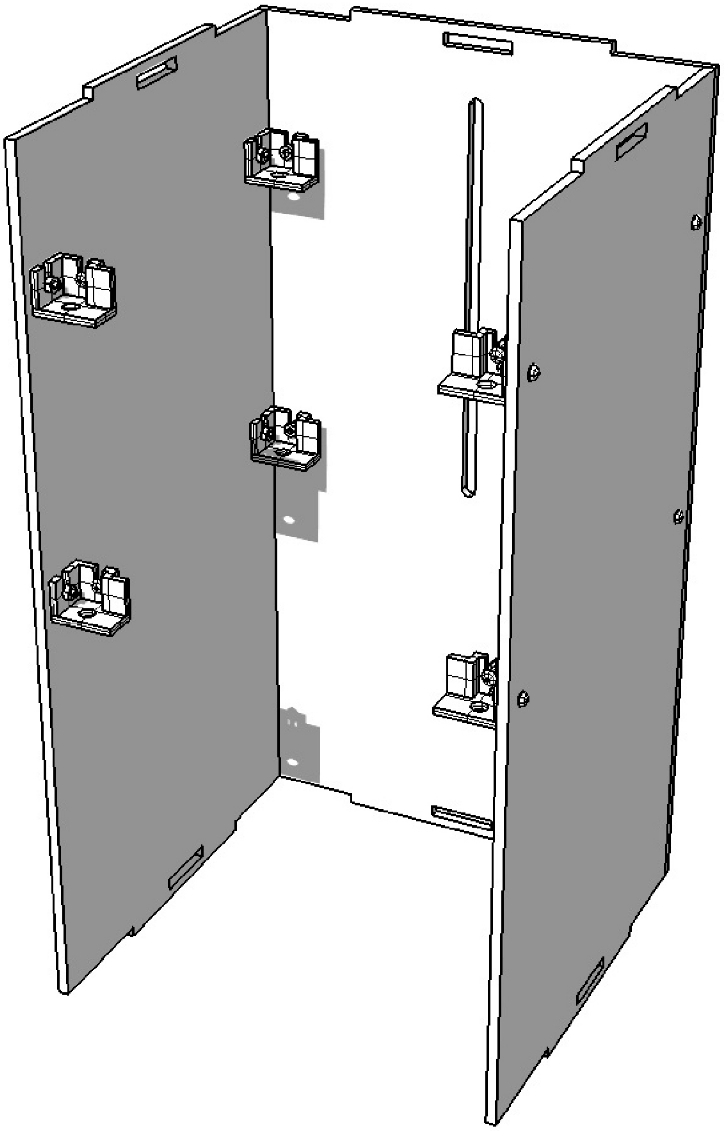

Final assembly view.

Step 3

With the vertical structure fastened, place T11 between the three vertical plates before placing T10 and fastening the screws.

Place the paper diffusor on top of T11 before use.

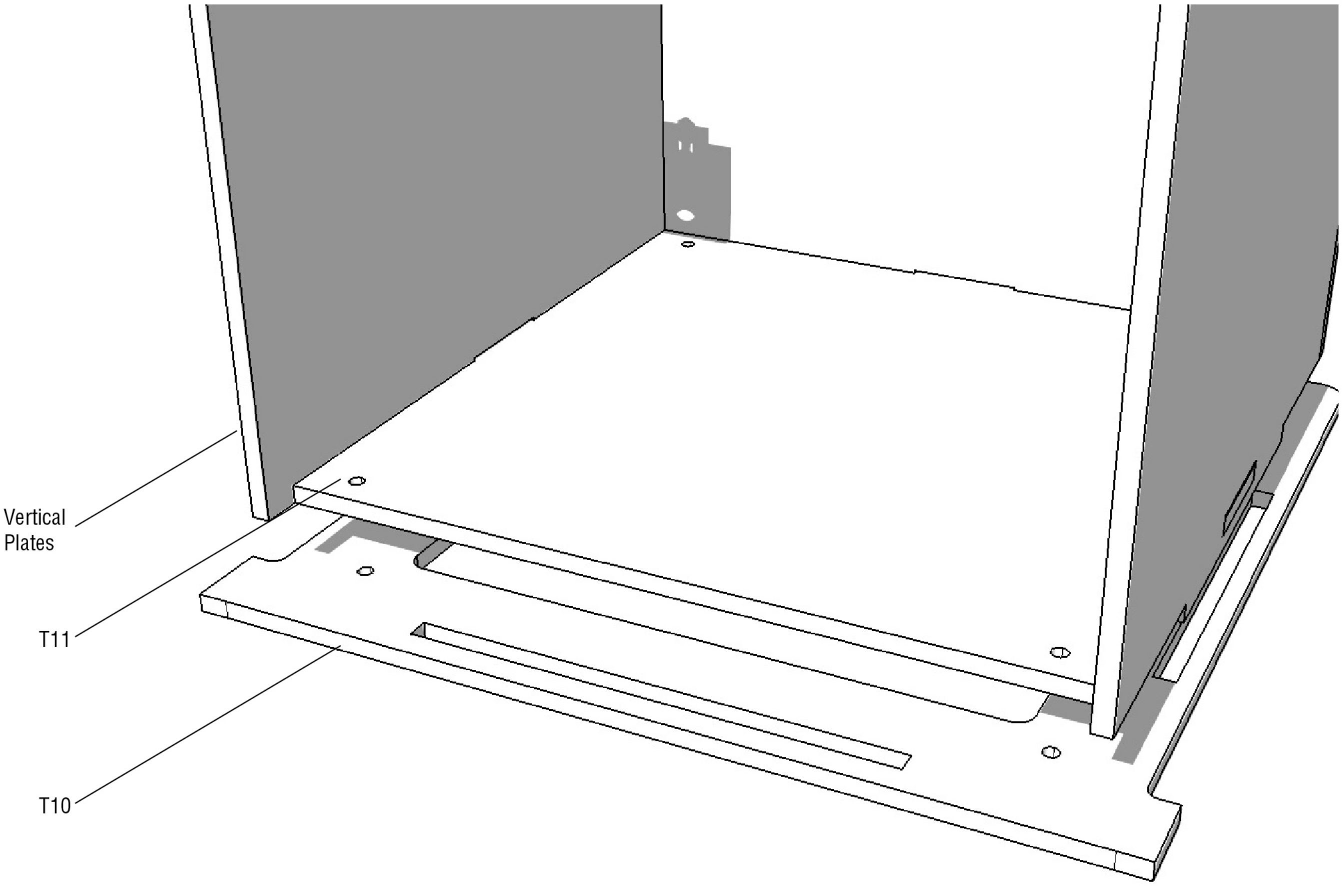

Final assembly view.

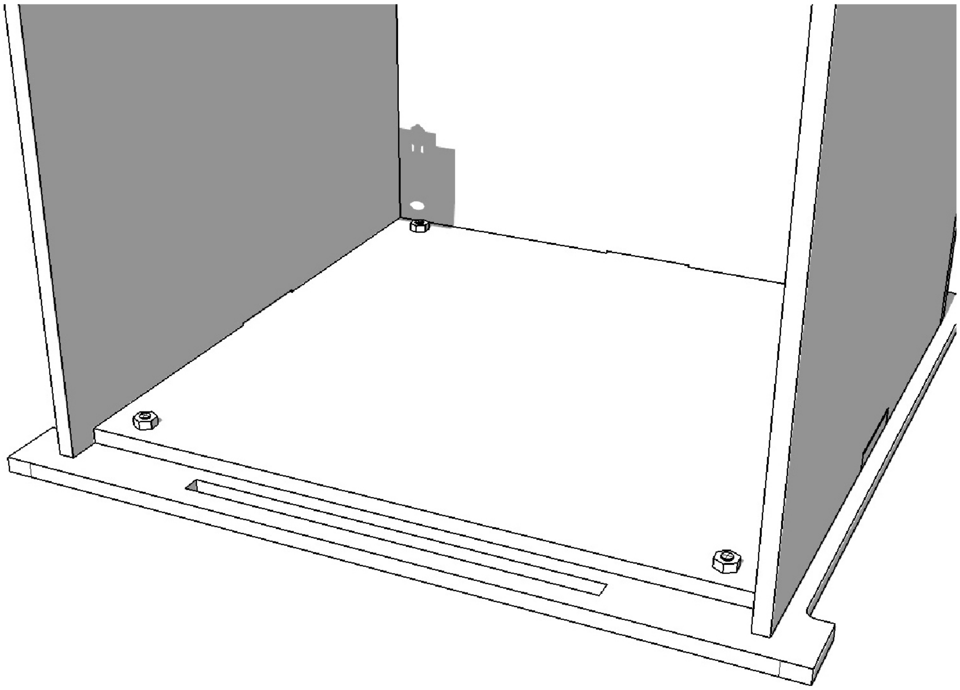

Step 4

Slide down the frontal plate (T9) until the screws fit in the spacers and tighten.

Make sure the front spacers are in the correct position (as in picture).

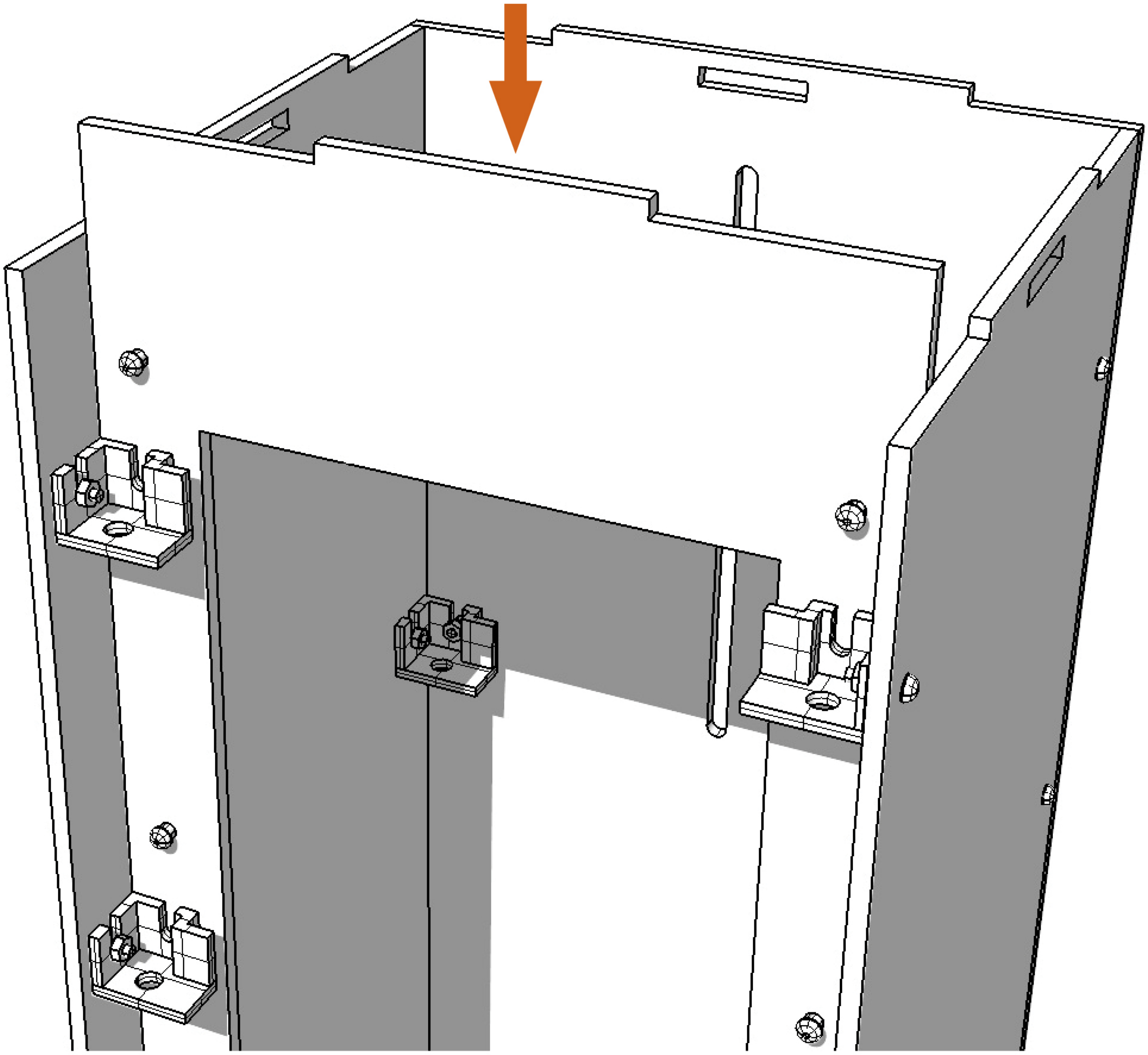

Slide down the back plate of the vertical movement bed. Must fit in the rail formed by the spacers.

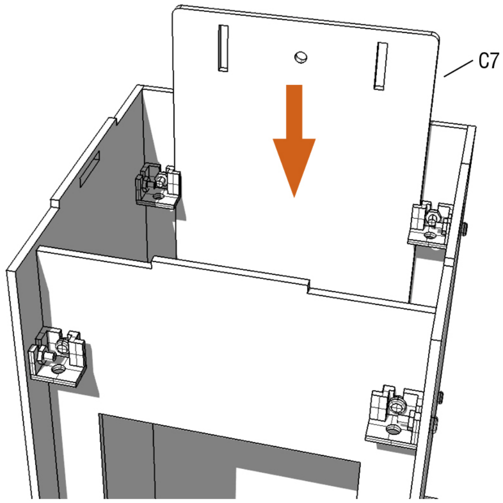

Step 5

Place T4 in the sloth of the vertical plates before putting the rest of the plates (T1, T2, T3).

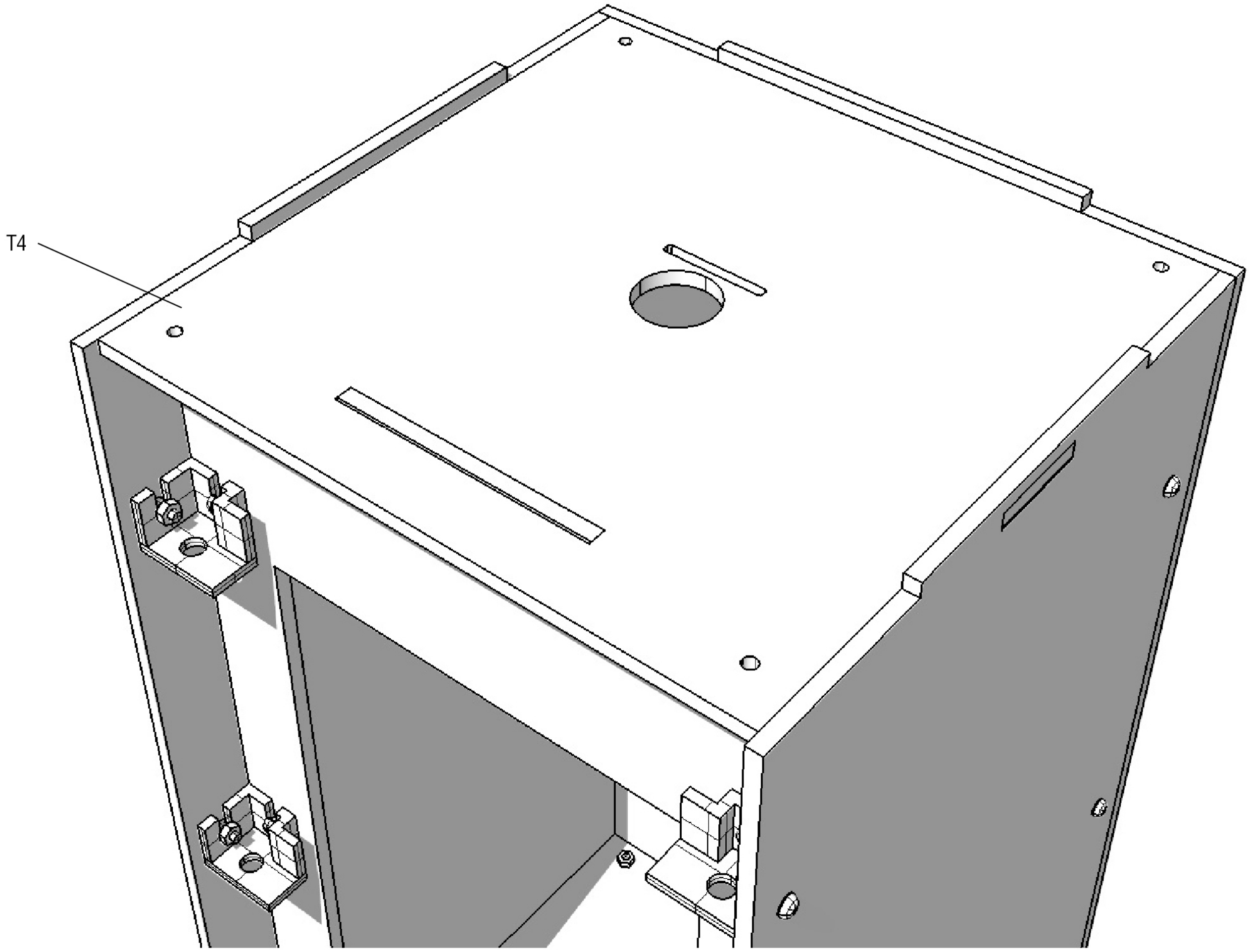

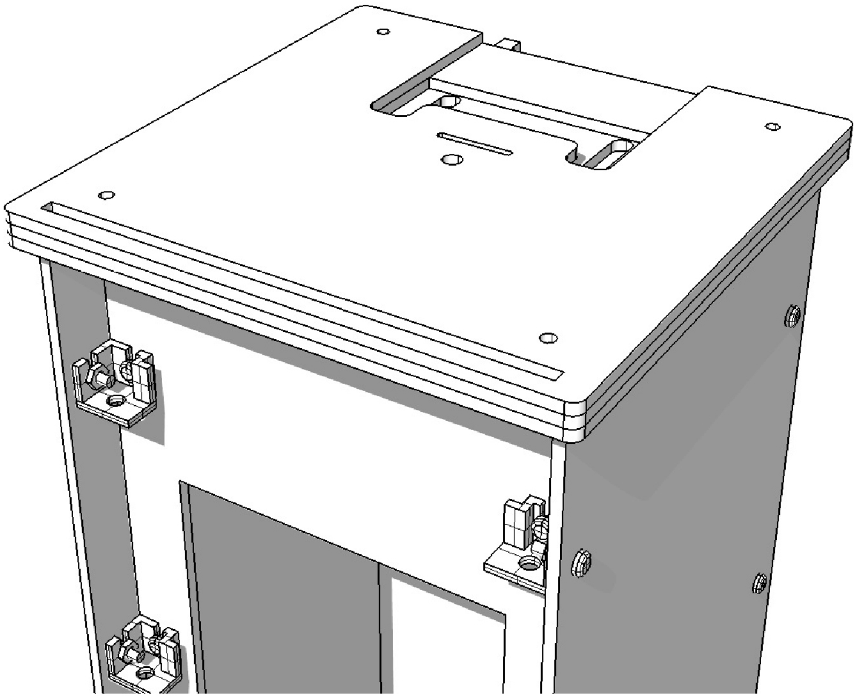

Final assembly view.

Fully Assembled Top Module Main Structure

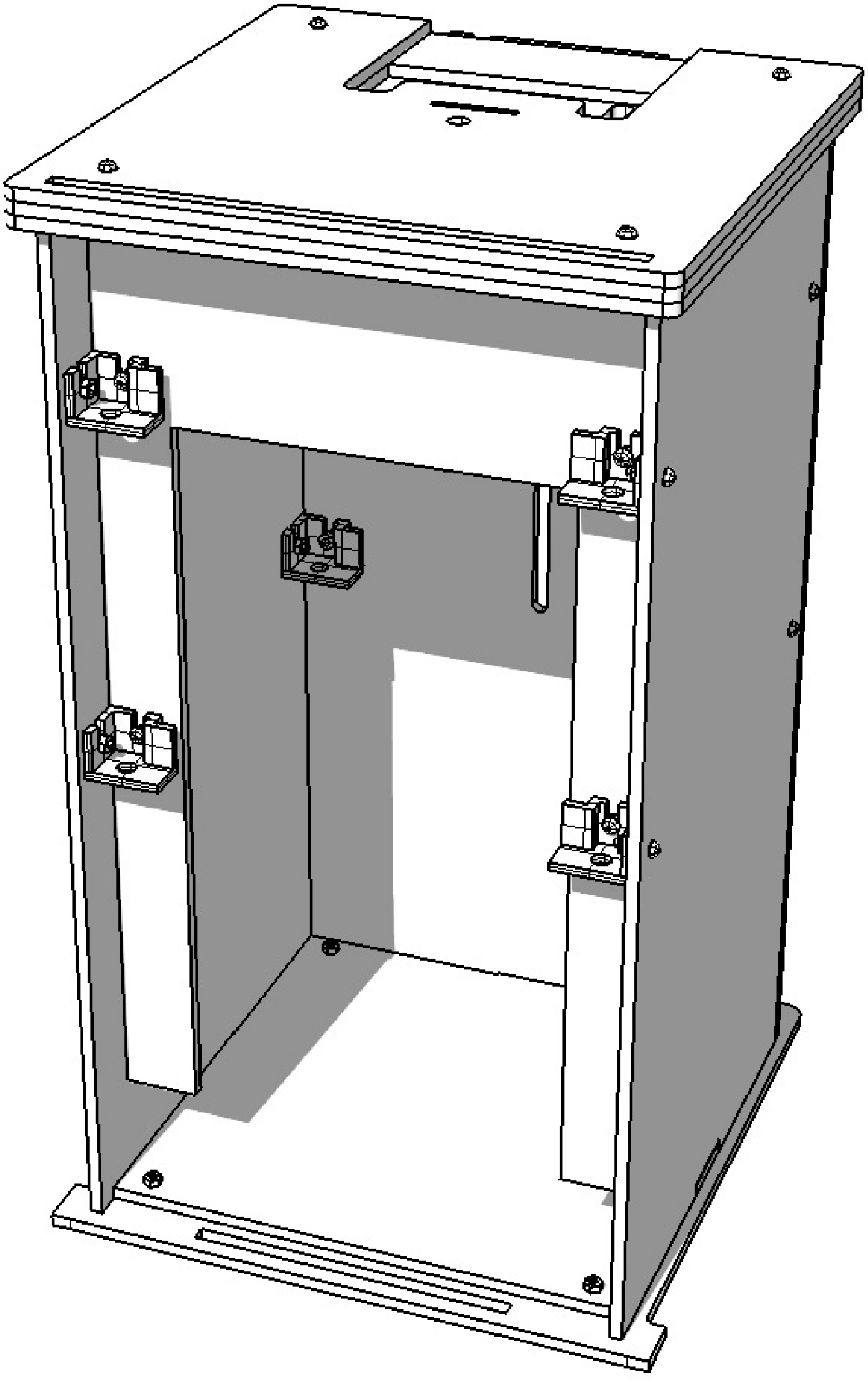

Bottom Module Explosion

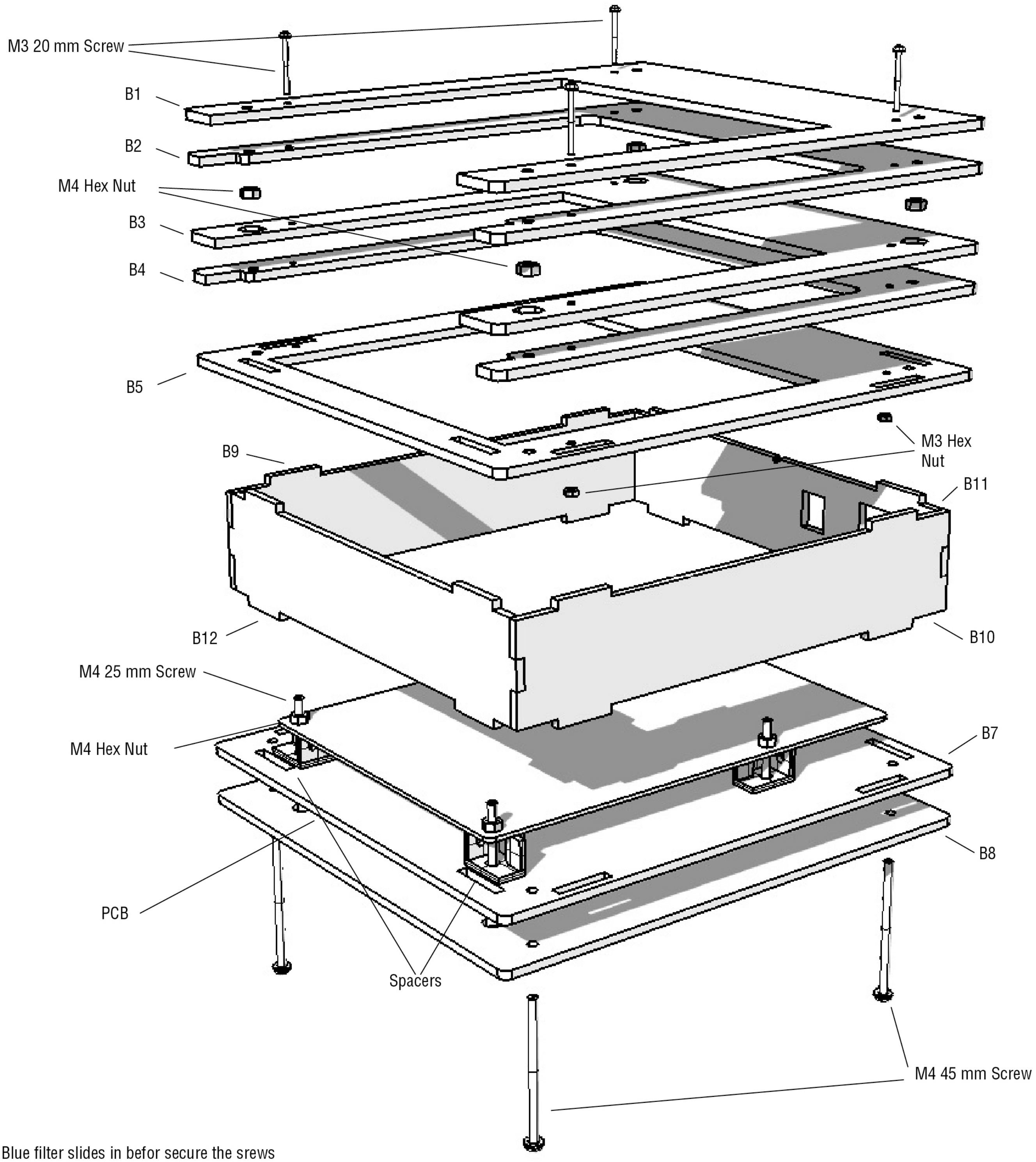

Blue filter slides in befor secure the srews

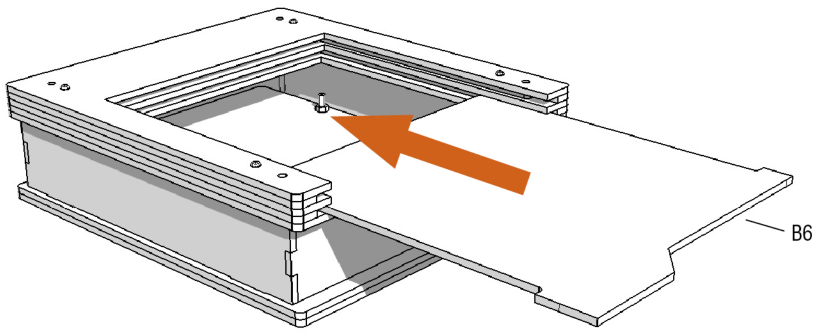

Bottom Module Parts

B1

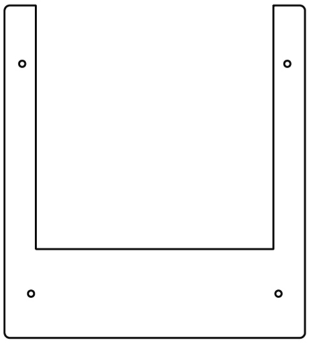

B2

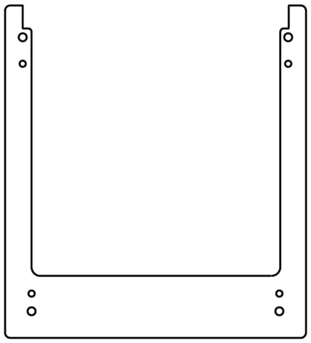

B3

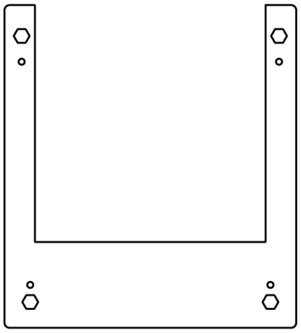

B4

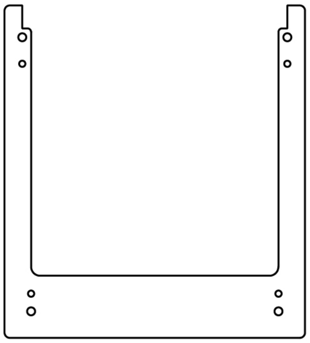

B5

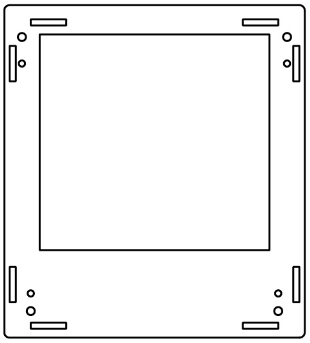

B6

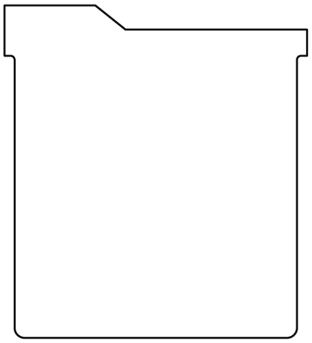

B7

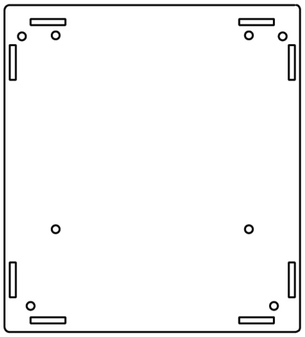

B8

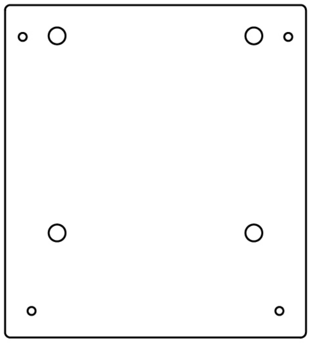

B9

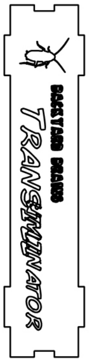

B10

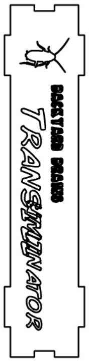

B11

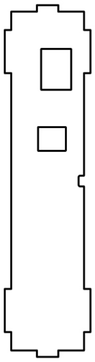

B12

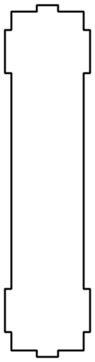

2 Spacers R and 2 Spacers L

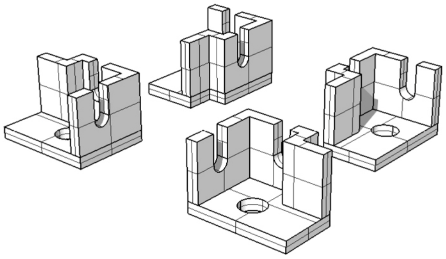

Screws and Nuts

4 M3 20 mm long screws and nuts for the top cover.

4 M4 45 mm long screws and nuts for the botom cover and main assembly.

4 M4 25 mm screws and nuts for the PCB mounting.

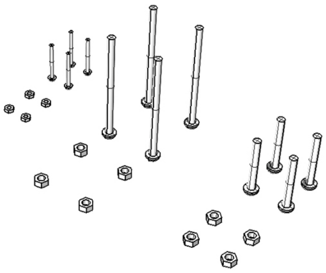

Top Module Assembly

Step 1

Assembly top cover. Put together B1, B2, B3, B4 and B5. Make sure you place the M4 Hex nut inbetween B2 and B3. You can place the parts upside down to make it easier.

Fasten with the 4 M3 20 mm screws and nuts.

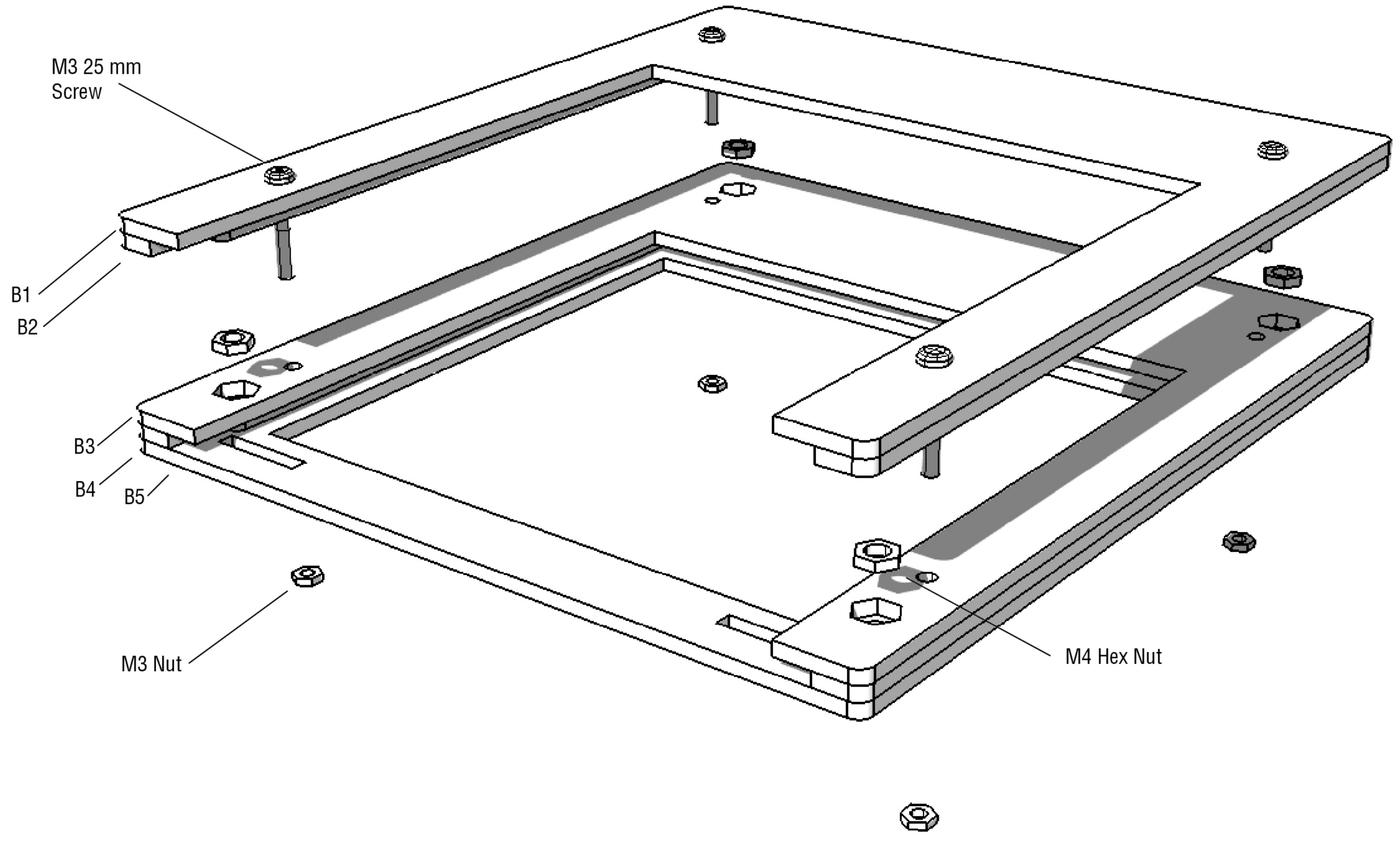

Final assembly view.

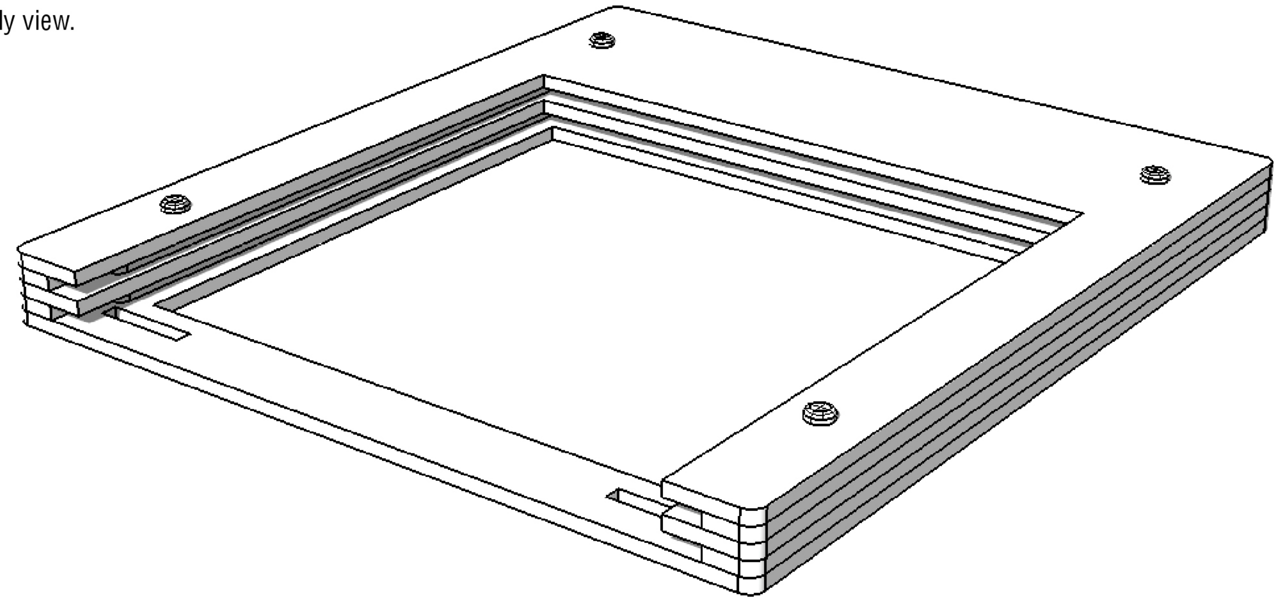

Step 2

Assembly B7 with the PCB using the 4 M4 22 mm screws and nuts
and 2 spacers R and 2 Spacers L as shown in the picture.

Make sure the front spacer are in the same position as in picture.

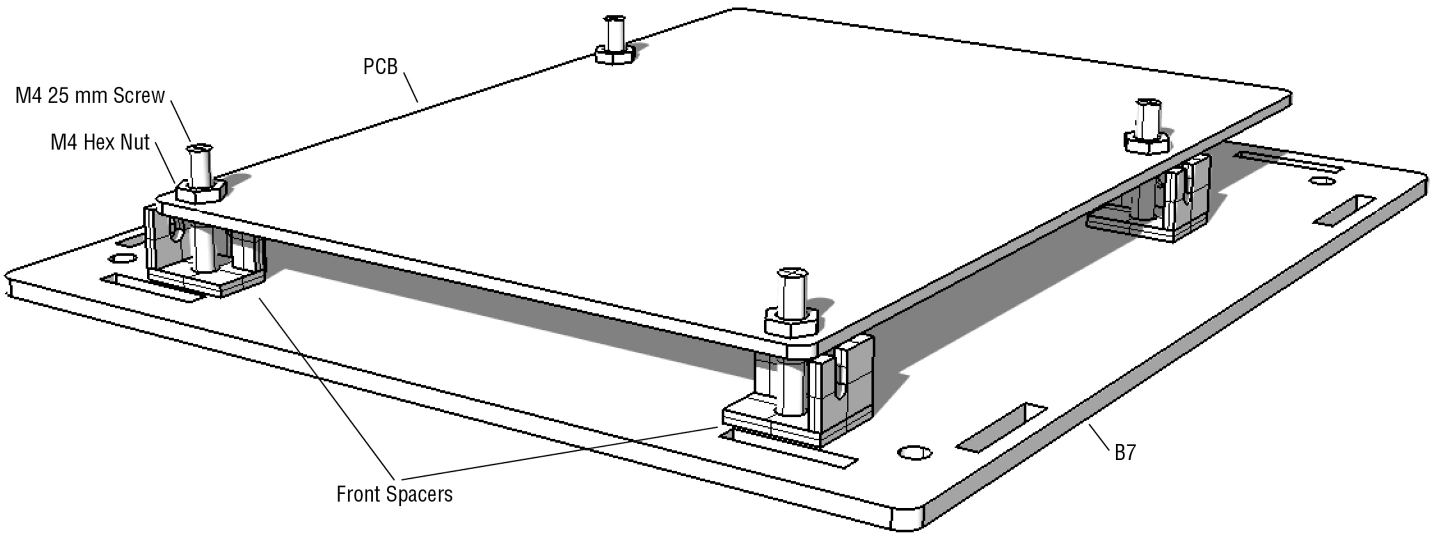

Front Spacers Position Detail

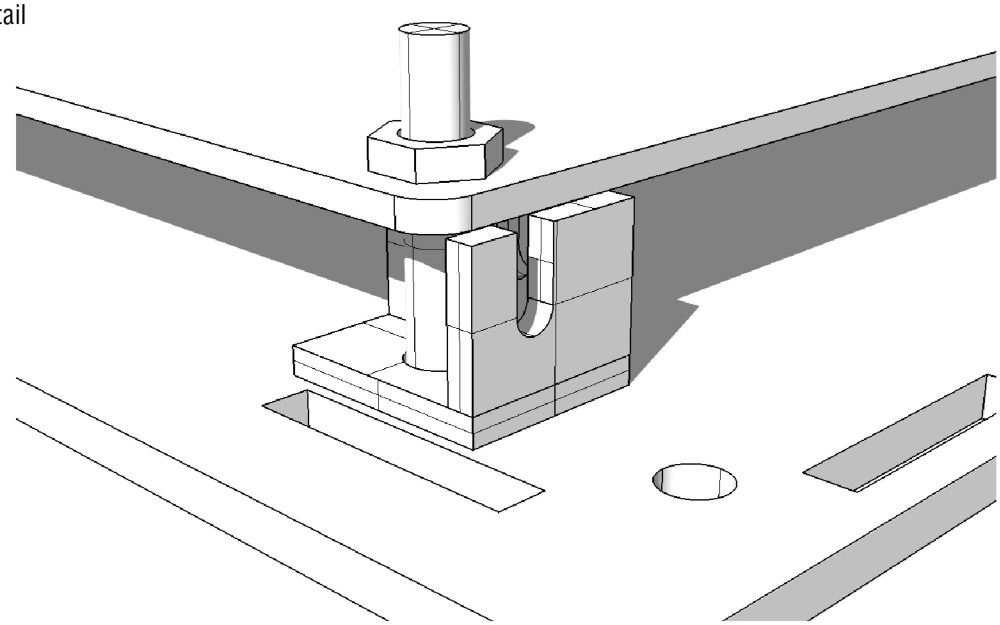

Step 3

Assembly the on/off switch by snaping it to the back plate (B11), then solder into the pcb.

Make sure the cable for the digital control goes through the little sloth on top of B11.

With all the electronics in place, put the rest of the plates in (B9, B10 and B12).

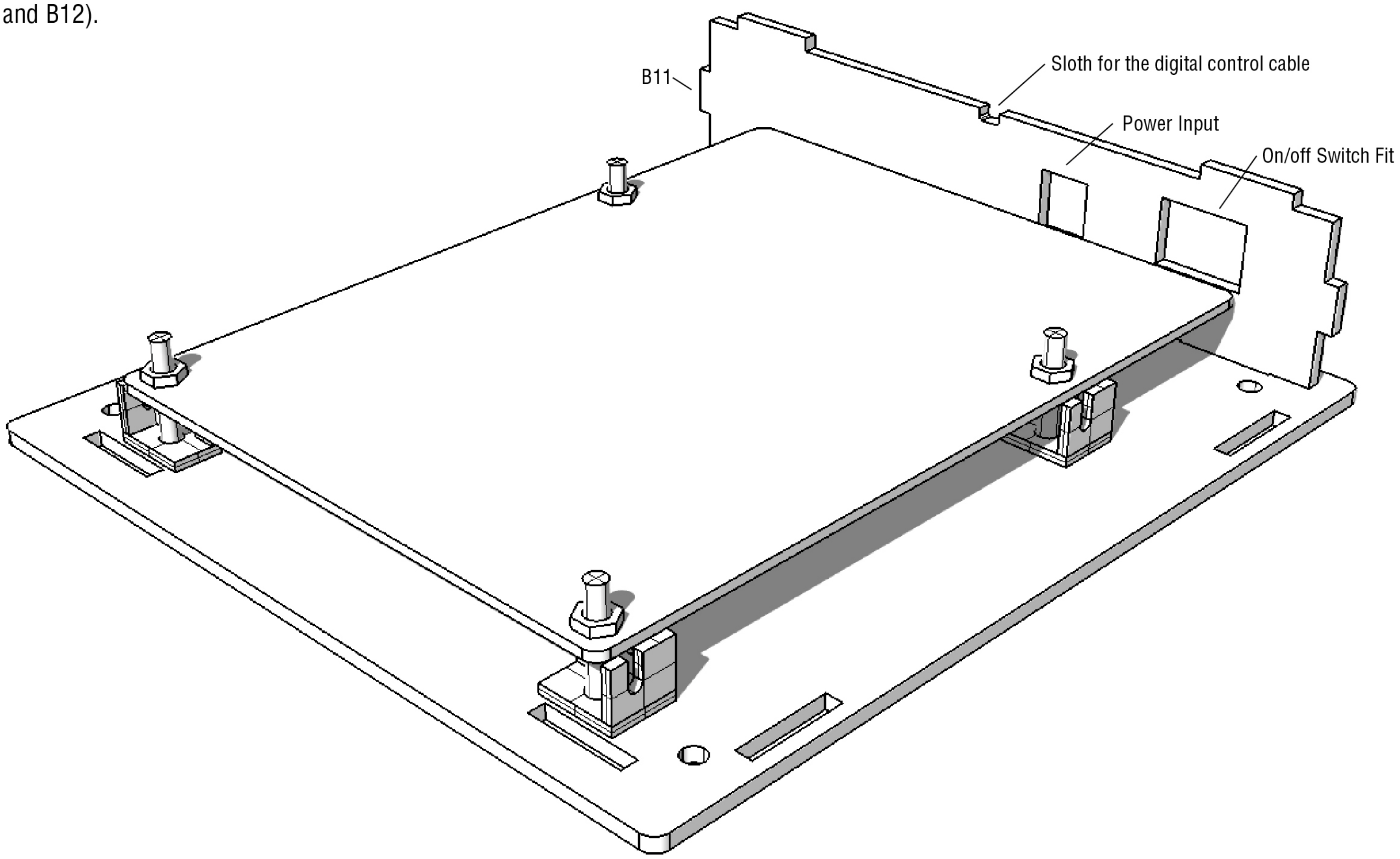

Final Assembly View

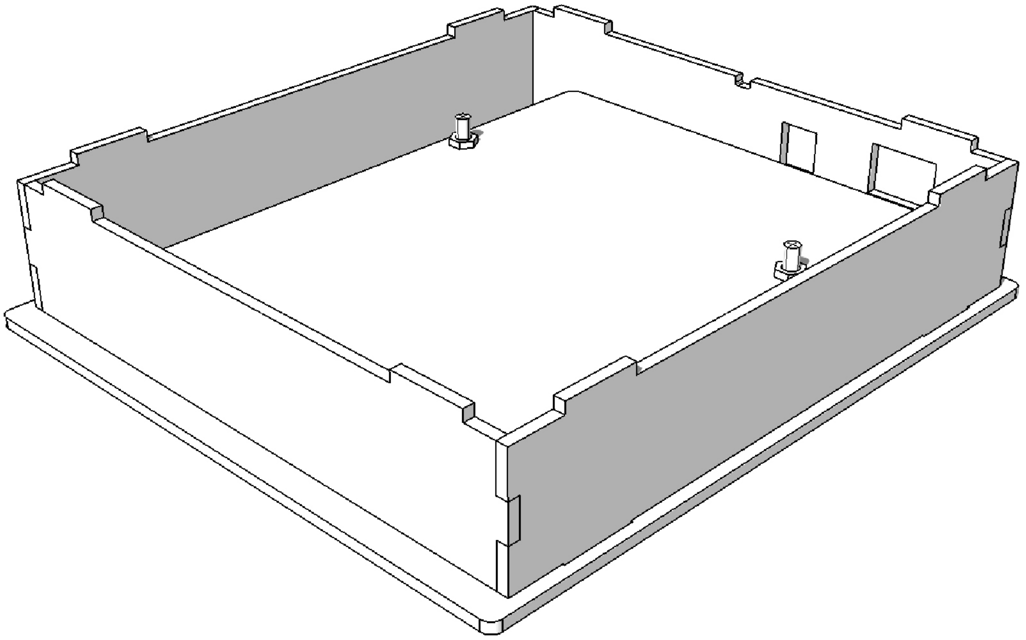

Step 4

Assembly the top cover in the lateral plates. fasten with the 4 M4 50 mm long screws in the nuts inside the top cover. Place B8 on the bottom before puting the screws.

Don’t tighten the screws too hard, you will need them loose to slide in the top module and the filter.

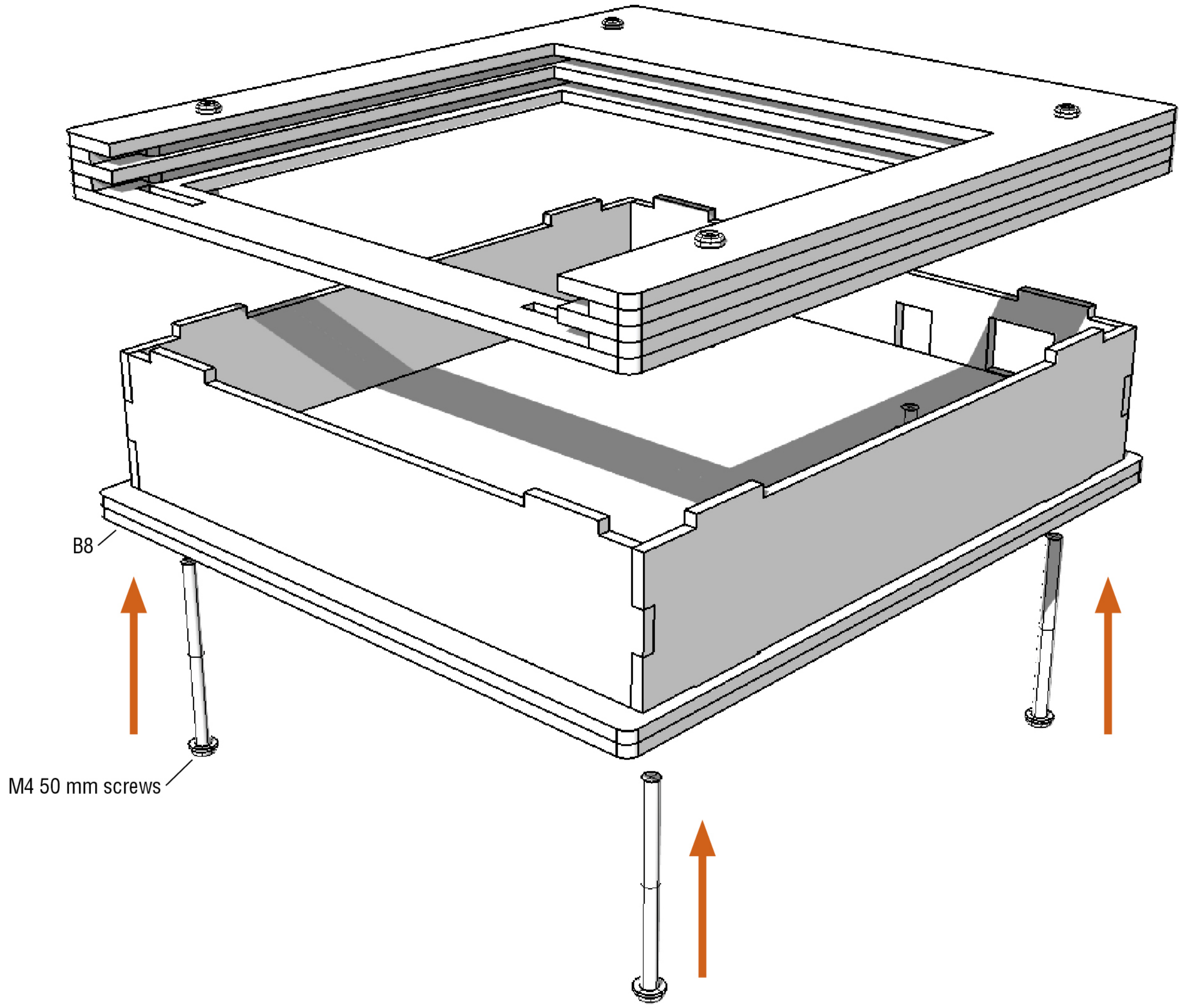

Final Assembly View

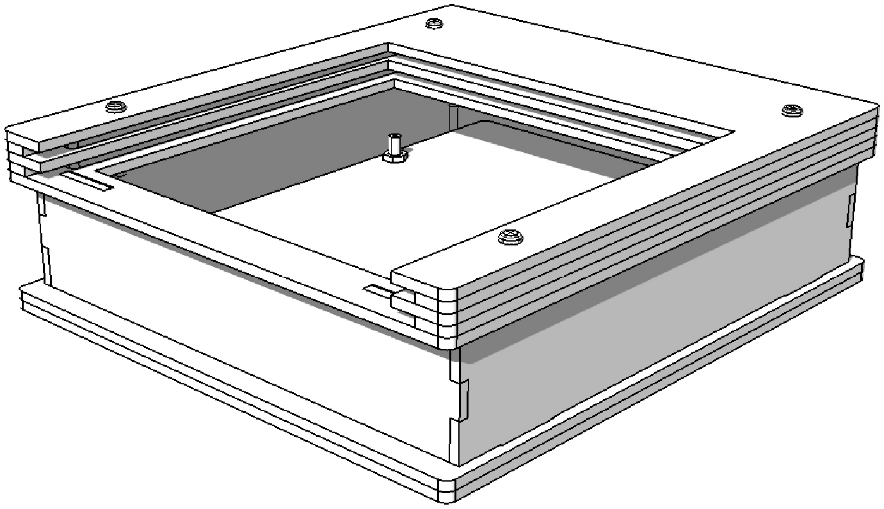

Step 5

Slide in the blue filter (B6) on the bottom sloth.

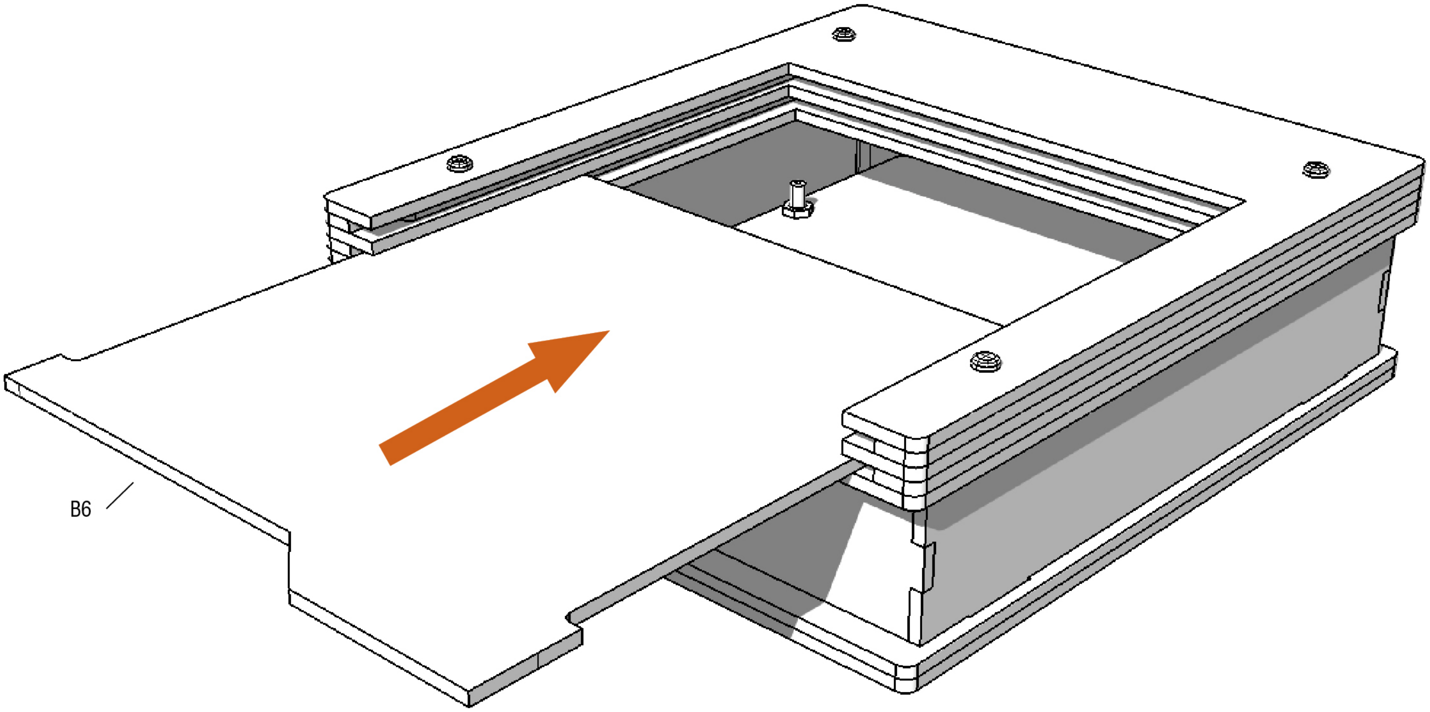

Top Module and Bottom Module Assembly

Slide the top module in the bottom module sloths.

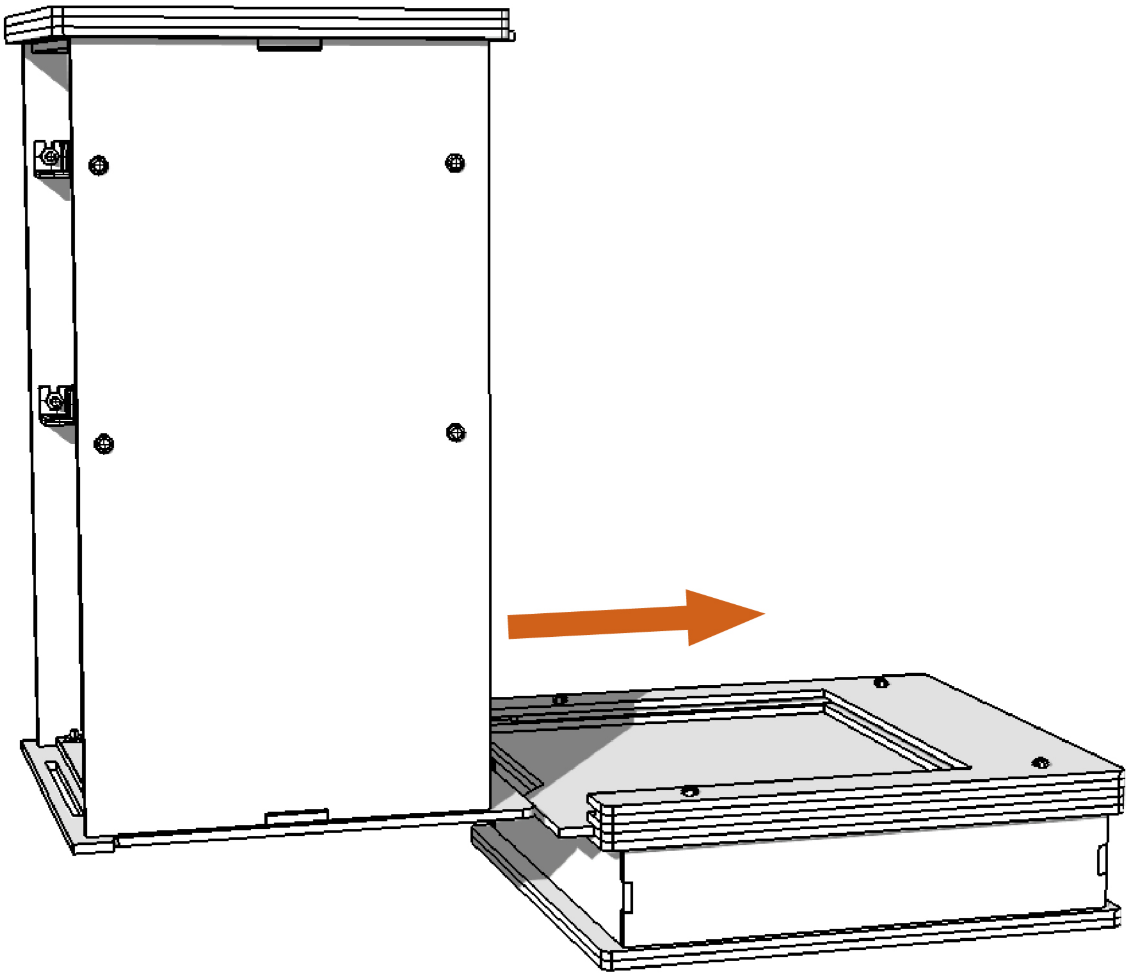

Slide the top module in the superior sloth. Push carefully until the top module fit all the way into the bottom module.

If it is too tigth, loose the top screws or the bottom screws.

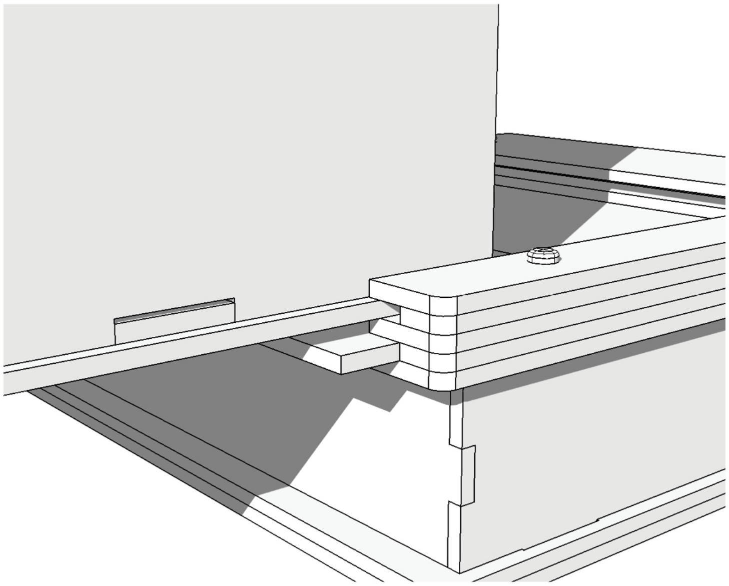

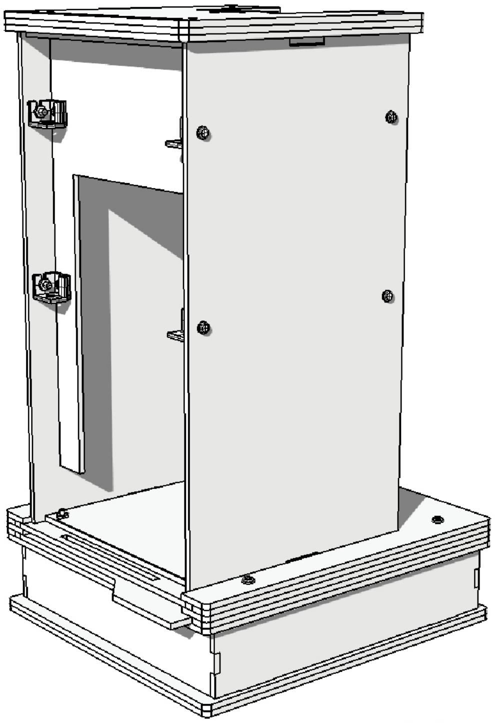

Final assembly view.
