# Supplementary material for: Low cost and open source multi-fluorescence imaging system for teaching and research in biology and bioengineering
Source: PLoS One. 2017 Nov 15;12(11):e0187163. doi: 10.1371/journal.pone.0187163 (PMC5687719; doi:10.1371/journal.pone.0187163)

Raspberry Pi Adaptor Assembly

The mounting holes on the Raspberry are sligthly smaller than the screws. Use one of the screws to make it bigger by screwing it in and then out of the board.

Place the 3 plates together R1, R2, R3, R4, R5, R6 and R7 with the Raspberry Pi, as shown in the picture.

Fasten with the screws and nuts.

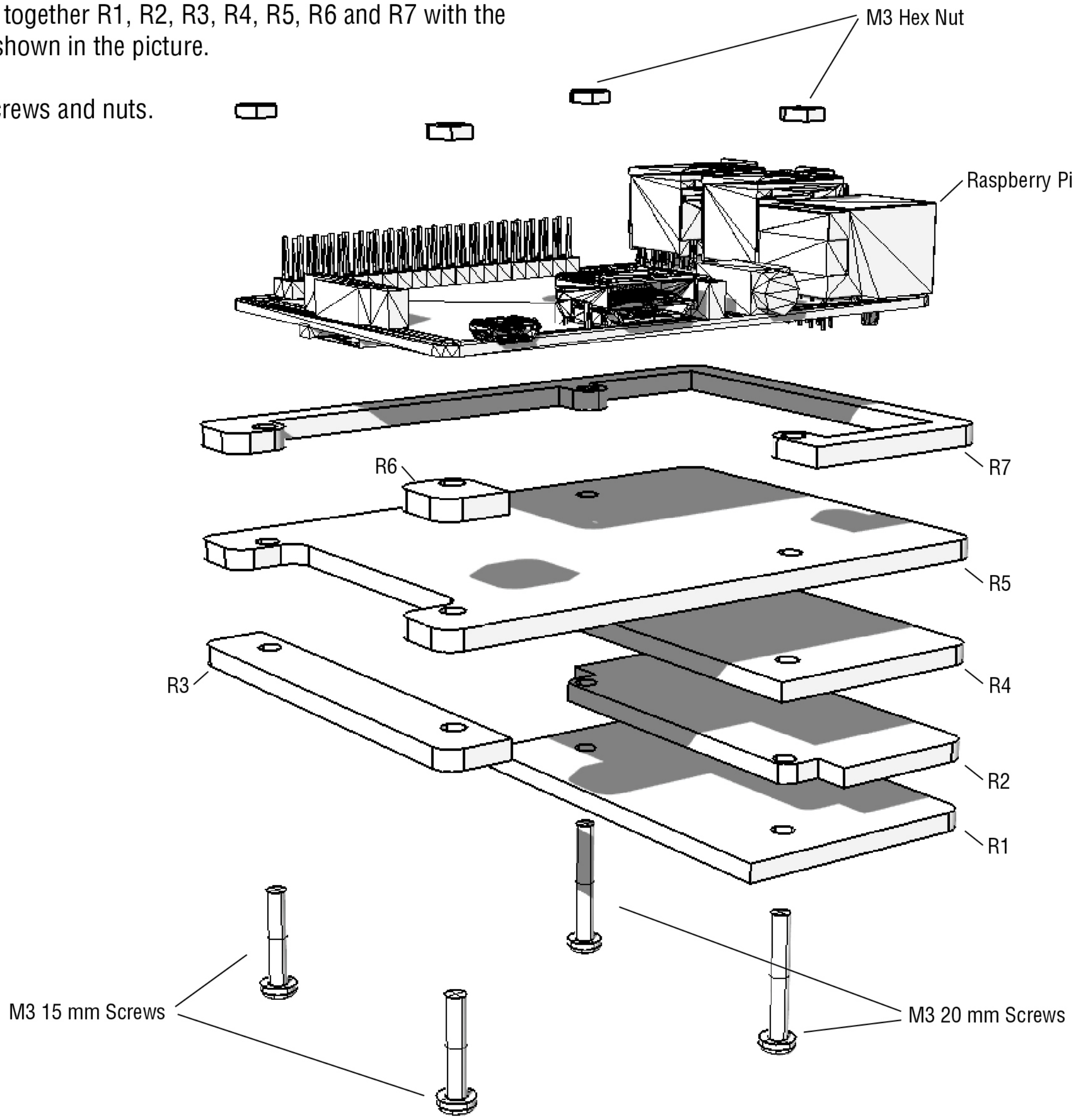

Mounting of the adaptor

Place the adaptor on top of the top module and slide it forward until it's firmly attached.

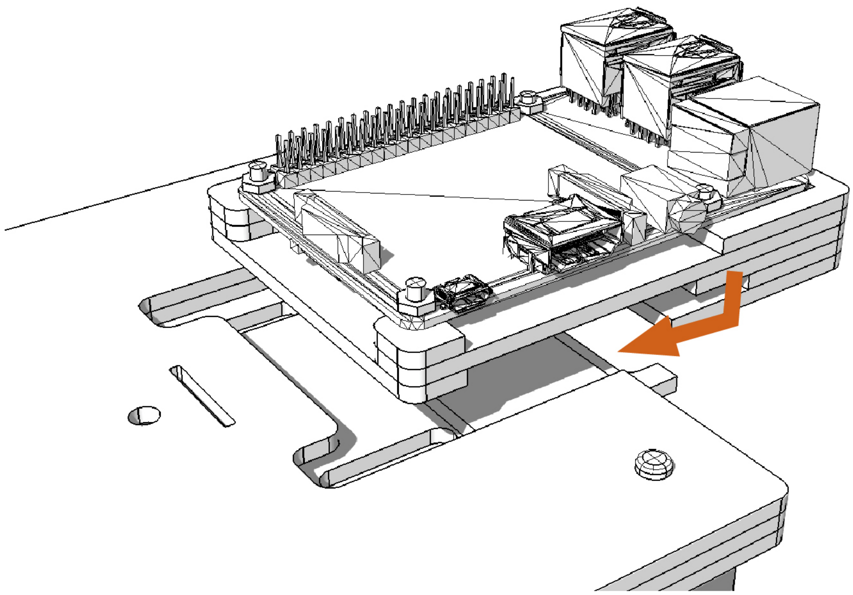

Raspberry Pi Adaptor Parts

R1

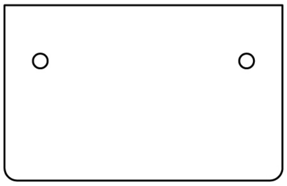

R2

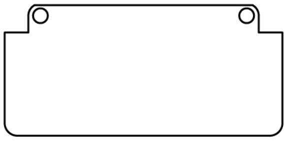

R3

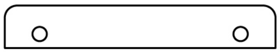

R4

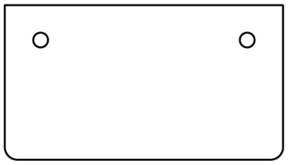

R5

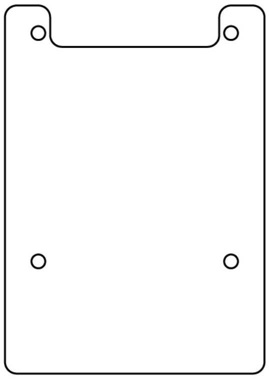

R6

R7

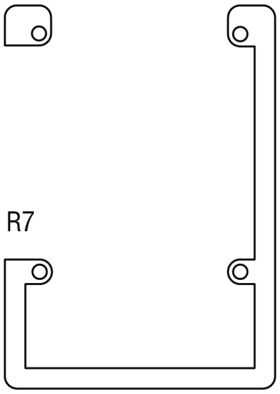

Screws and Nuts

2 M3 15 mm Screws and Nuts.
2 M3 20 mm Screws and Nuts.

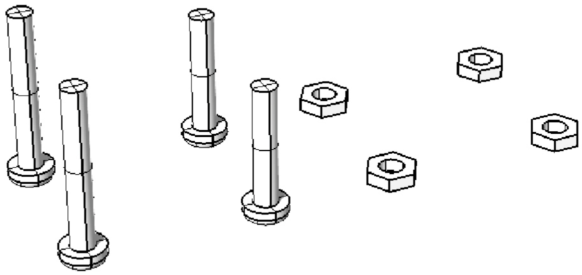

Supplement: S2 File — (PDF) [file pone.0187163.s004.pdf]
