# Supplementary material for: Low cost and open source multi-fluorescence imaging system for teaching and research in biology and bioengineering
Source: PLoS One. 2017 Nov 15;12(11):e0187163. doi: 10.1371/journal.pone.0187163 (PMC5687719; doi:10.1371/journal.pone.0187163)

Camera Bed Assembly

Step 1

Place C2, C3, C4, C5 and C6 together and slide in C8 and C9 (with the M4 Nut inside).

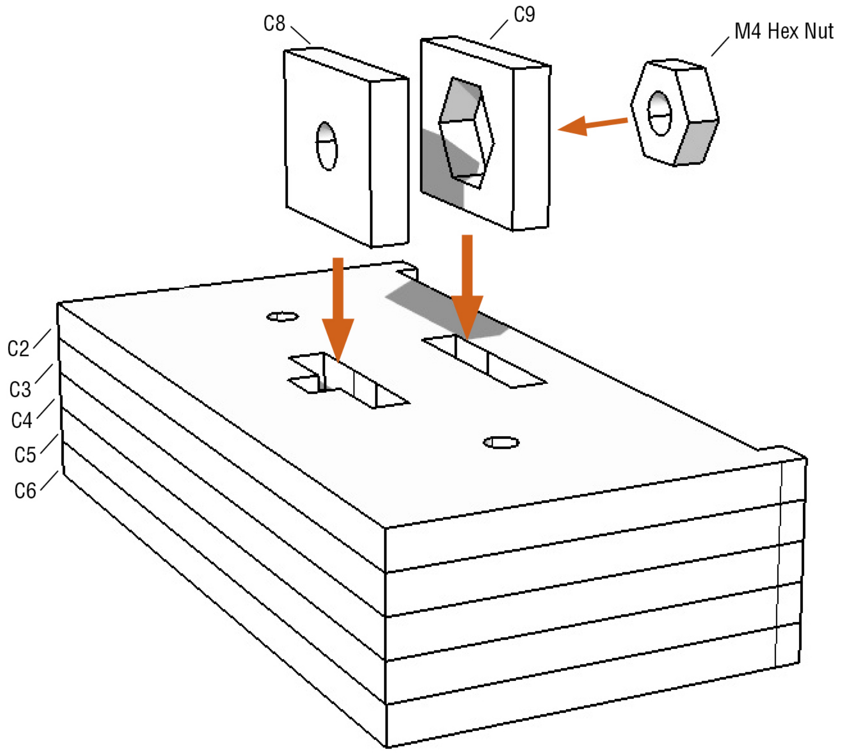

Step 2

Use C1 to cap all the other pieces and fasten with the two M4 22 mm screws and nuts.

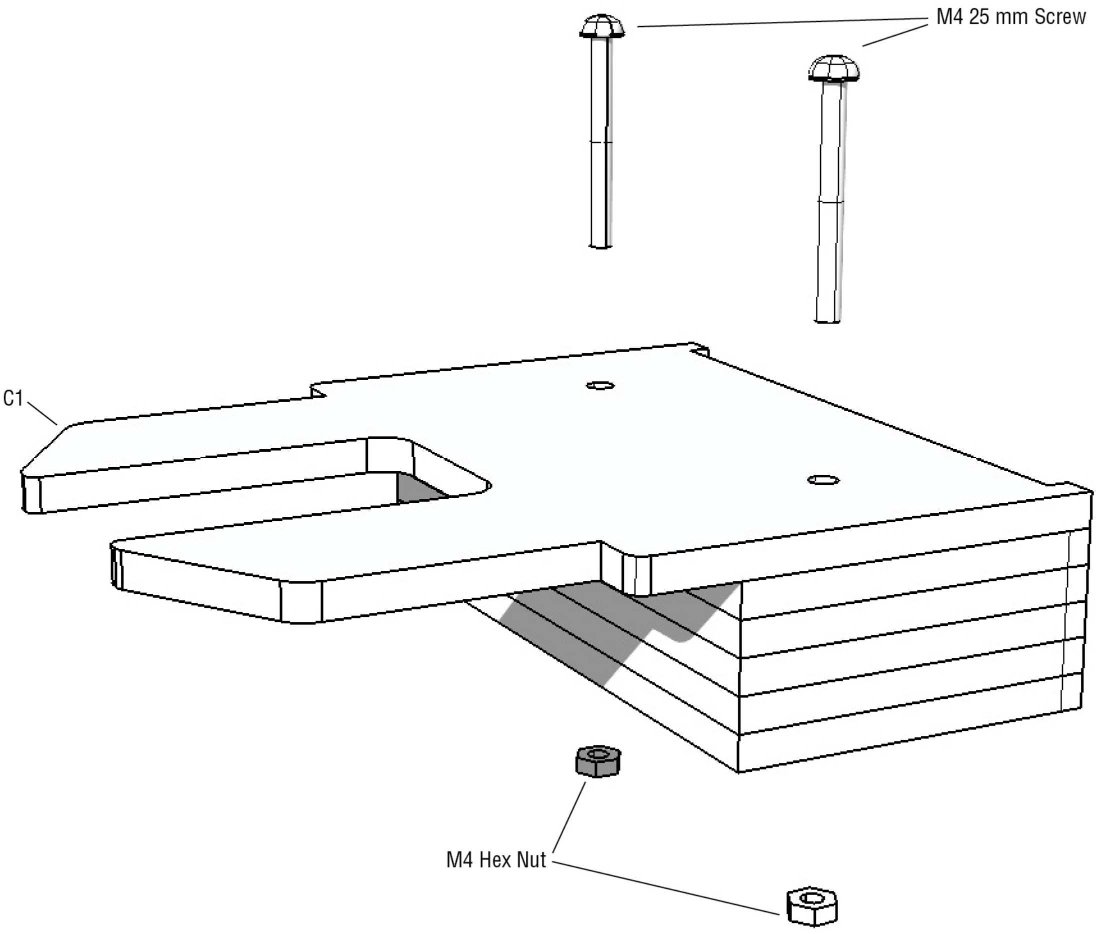

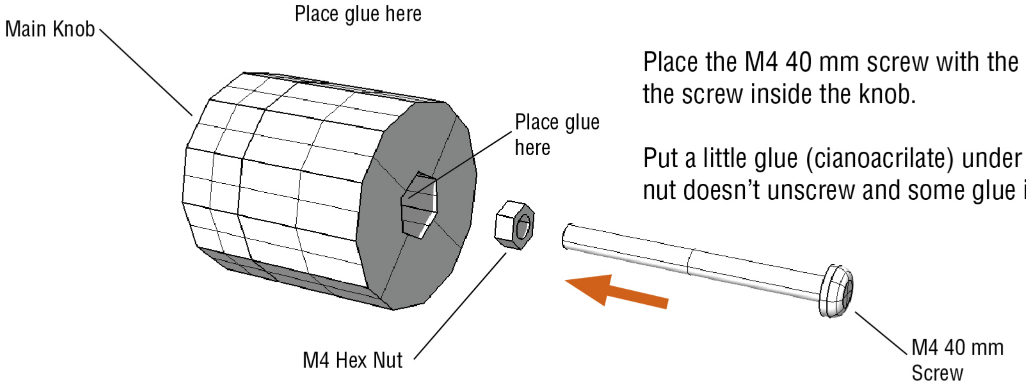

Place the M4 40 mm screw with the nut all the way to the head of the screw inside the knob.

Put a little glue (cianoacrilate) under the head of the screw so the nut doesn't unscrew and some glue inside the knob to fix the screw.

Step 3

Mount the main knob with the M4 40 mm long screw and nut as in picture.

First, place the back plate (C7) by sliding it in the spacers.

Then, use a screwdriver to fasten the main knob to the vertical bed.

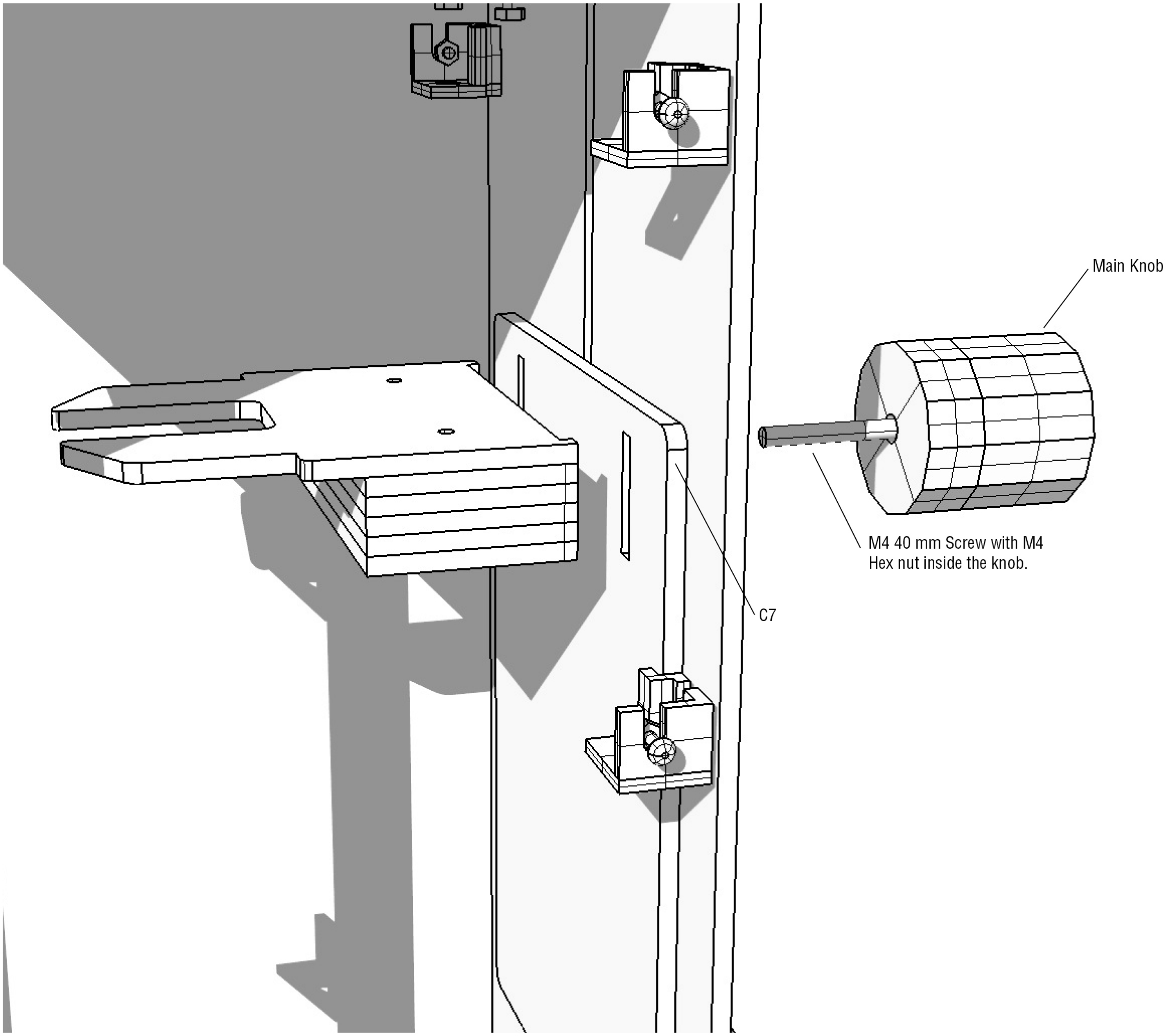

Camera Bed Explosion

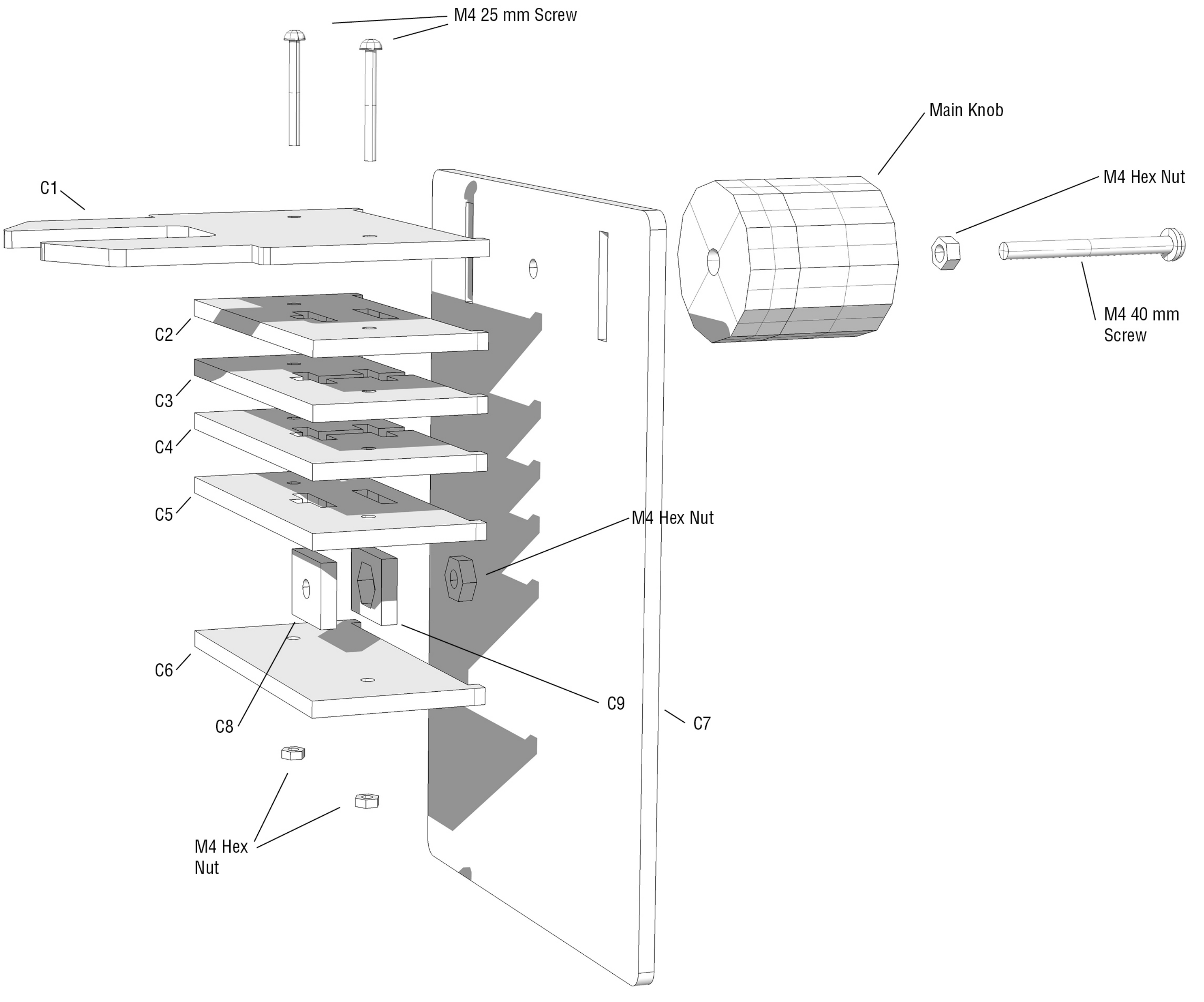

Camera Bed Parts

C1

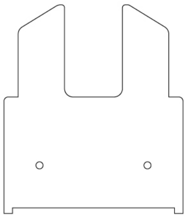

C2

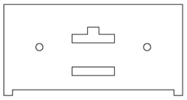

C3

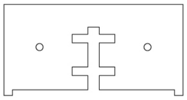

C4

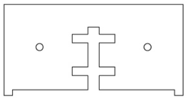

C5

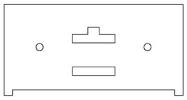

C6

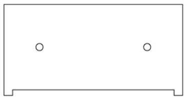

C7

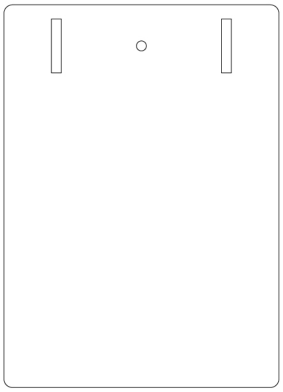

C8

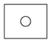

C9

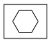

Main Knob

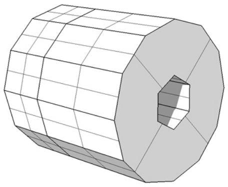

Focus Knob

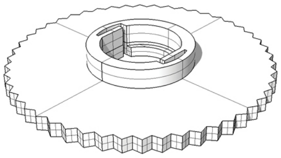

C10

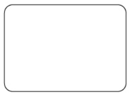

Screws and Nuts

2 M4 25 mm long screws and nuts for the bed.

1 M4 40 mm long screw and nut for the main knob.

1 M4 Hex Nut to fix the bed to the knob.

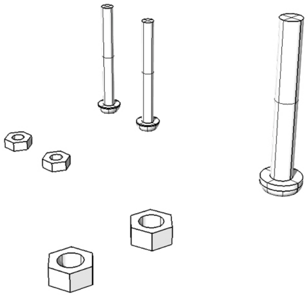

Camera Adaptor Mount

Mount the focus knob to the camera as in the picture, press the camera lens down on the knob until it fits. Use a drop of glue to fix.

Mount the camera on the adaptor by sliding it in.

Focus Knob and Camera Module Mount

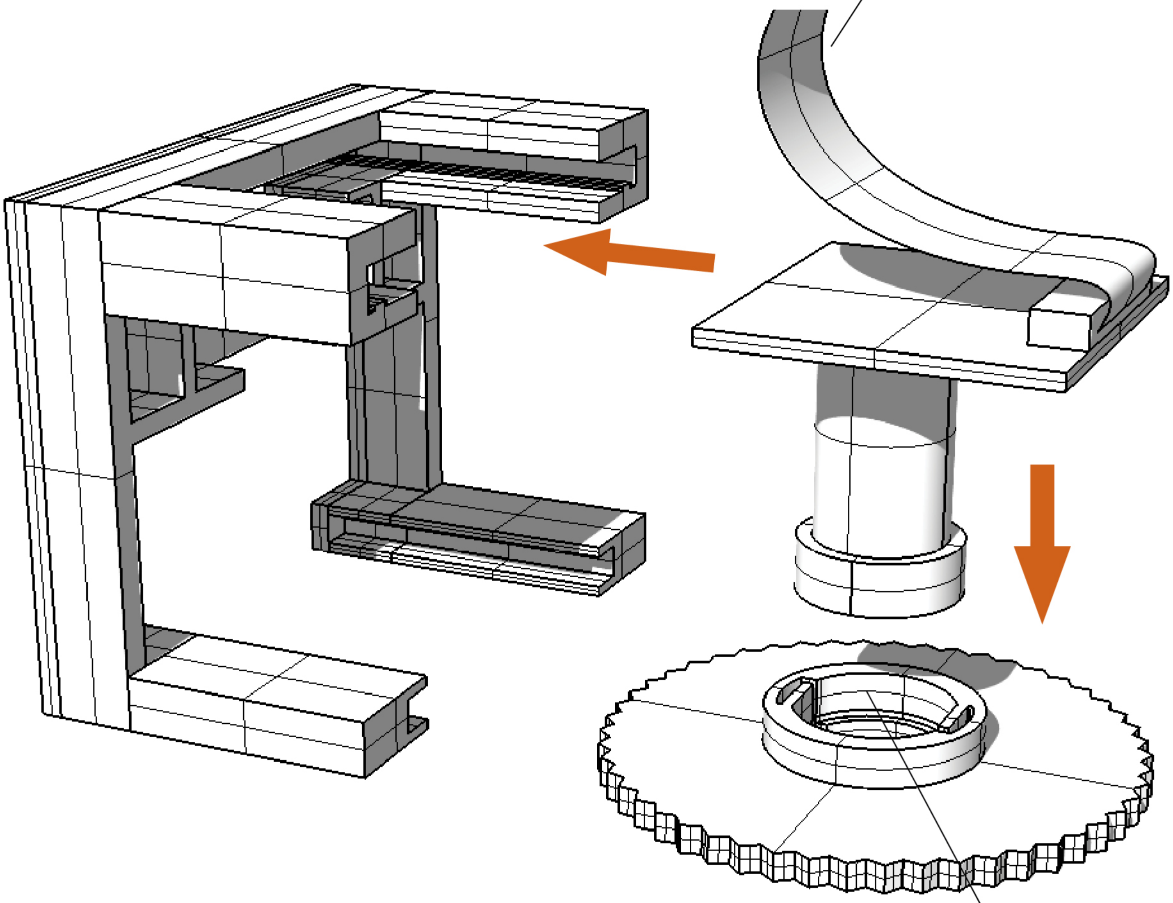

Slide in the amber filter (C10) in the camera adaptor as shown in the picture.

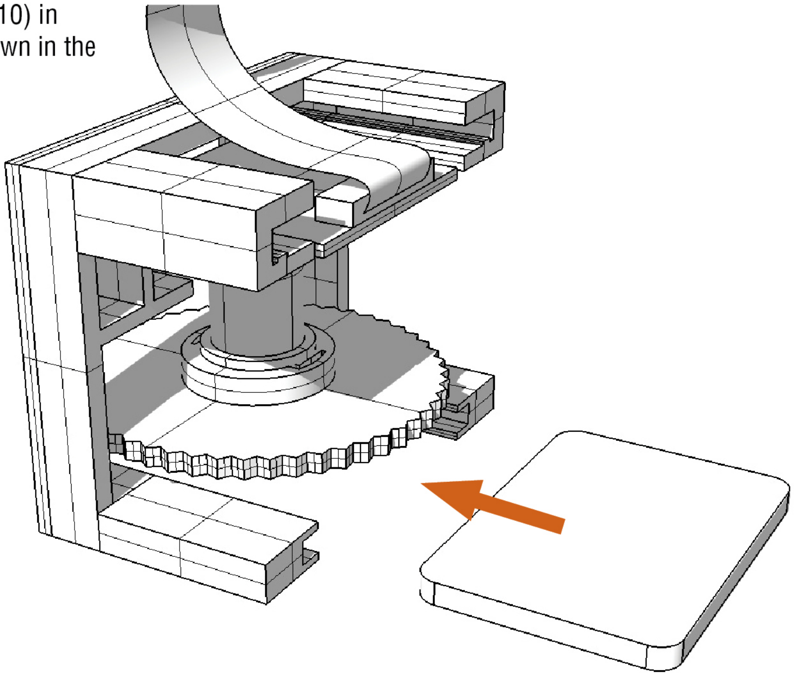

Camera Adaptor Final Mounting

Slide the camera adaptor in the vertical movement bed until fits firmly.

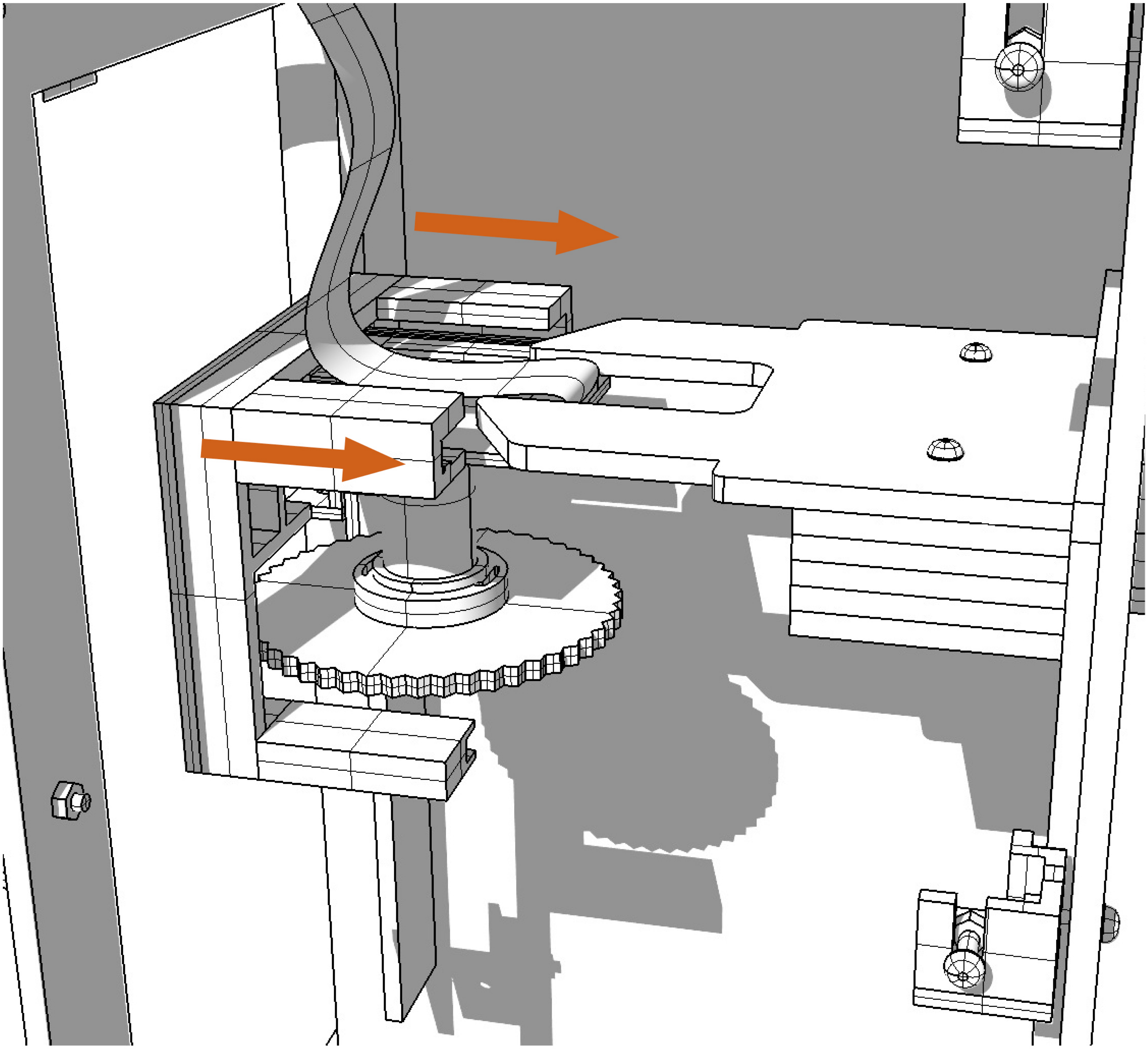

Supplement: S3 File — (PDF) [file pone.0187163.s005.pdf]
