# Supplementary material for: Low cost and open source multi-fluorescence imaging system for teaching and research in biology and bioengineering
Source: PLoS One. 2017 Nov 15;12(11):e0187163. doi: 10.1371/journal.pone.0187163 (PMC5687719; doi:10.1371/journal.pone.0187163)
Supplement: S5 File — (PDF) [file pone.0187163.s007.pdf]

Fluorescence maths

September 29, 2017

0.0.1 Fluorescent signal maths analysis for timelapse images

Intensity signal $I_s(t)$ - or pixel value - can be decoupled on two terms: the signal given by the biological system $I_b(t)$ and the background signal given by the media $I_m(t)$.

$$I_s(t) = I_b(t) + I_m(t) \tag{1}$$

It is possible to obtain the background intensity directly from the data and get rid of it by simple substraction (assuming independence between them), focusing on the intensity given by the biological system.

$$I_b(t) = I_s(t) - I_m(t) \tag{2}$$

This signal intensity of interest ("$I(t)$") is given by the amount of fluorescence produced by bacteria. Then, it is possible to write the next expresión to represent it.:

$$I(t) = V(t) \cdot F_v \tag{3}$$

where $V(t)$ is the volume of bacteria on each space and time point, and F_v is the fluorescence emited by each unit of volume. As there are not precise measures of the colony volume or its absorbance at each point, it would be useful to make some simplifications. By assuming an homogeneus thickness along the colony (say h), then:

$$I(t) = V(t) \cdot F_v \rightarrow I(t) = A(t) \cdot h \cdot \frac{F_A}{h} \rightarrow I(t) = A(t) \cdot F_A \tag{4}$$

where $A(t)$ is the area of bacteria on each time point and F_A is the amount of fluorescence emited per unit of area (pixel)

This last term could be assumed as a linear relation with the amount of fluorescent protein on each unit of area $\left([FP] = \frac{FP}{Area}, \alpha_F = \text{fluorescence produced per unit of [FP]}\right)$:

$$F_A = a_F \cdot [FP] + b \tag{5}$$

Then, the following expression is obtained for the intensity:

$$I(t) = A(t) \cdot F_A = A(t) \cdot a_F \cdot [FP] + A(t) \cdot b \tag{6}$$

where the first term is given by the fluorescent protein amount on each cell and the second term is the bacterial colony autofluorescence.

Dividing by the area $A(t)$ it's is possible to obtain an expression for the mean fluorescent intensity per area:

$$\frac{I(t)}{A(t)} = a_F \cdot [FP] + b \tag{7}$$

By taking the derivative over time, it's get the expression:

$$\frac{d(I/A)}{dt} = a_F \cdot \frac{d[FP]}{dt} \tag{8}$$

It is possible to relate the above with the dynamics of the colony protein expression. Taking simple models for transcription and traduction rate, respectively:

$$\frac{d[m]}{dt} = K_m(t) - \delta_m[m] - \mu[m] \tag{9}$$

$$\frac{d[P]}{dt} = K_p(t) \cdot [m] - \delta_p[P] - \mu[P] \tag{10}$$

where $K_m(t)$ and $K_p(t)$ are the transcription and traduction rates, δ is the degradation rate and μ is the growth rate (being the last term the dilution one).

Under the assumptions of $\frac{d[m]}{dt} \approx 0$ (and dilution term neglected) $\rightarrow [m] = \frac{K_m(t)}{\delta_m}$, and taking $\delta_p \approx 0$, the protein dynamics becomes:

$$\frac{d[P]}{dt} = K_p(t) \cdot \frac{K_m(t)}{\delta_m} - \mu[P] \tag{11}$$

by grouping the protein expression term, it becomes:

$$\frac{d[P]}{dt} = K_e(t) - \mu[P] \tag{12}$$

Using this model to represent the fluorescent protein dynamics, it's possible to use it on the mean fluorescence intensity expression developed (eq. 8):

$$\frac{d(I/A)}{dt} = a_F \cdot \frac{d[FP]}{dt} = a_F \cdot (K_e(t) - \mu[FP]) \tag{13}$$

$$\rightarrow \frac{d(I/A)}{dt} = K_F(t) - \mu \cdot a_F \cdot [FP] \tag{14}$$

where $K_F = a_F \cdot K_e$ is the flurescence intensity expression rate.

Finally, rearranging (14) and using (7):

$$K_F(t) = \frac{d(I/A)}{dt} + \mu \cdot \left(\frac{I}{A} - b\right) \tag{15}$$

As the colony autofluorescence is negligible it's possible to write:

$$K_F(t) = \frac{d(I/A)}{dt} + \mu \cdot \left(\frac{I}{A}\right) \tag{16}$$

To write an expression for the mean intensity signal (I/A) and for the growth rate (μ), it's possible to take the bacterial growth expression:

$$\frac{dV(t)}{dt} = \mu \cdot V(t) \tag{16}$$

Which under assumption of constant thicknes it gets:

$$\frac{dA(t)}{dt} = \mu \cdot A(t) \tag{17}$$

$$\rightarrow \mu(t) = \frac{1}{A(t)} \cdot \frac{dA(t)}{dt} \tag{18}$$

by taking a logistic area growth model:

$$A(t) = \frac{A_{max}}{1 + e^{-\mu_{max}(t-t_0)}} \tag{19}$$

$\mu(t)$ gets:

$$\mu(t) = \frac{\mu_{max}}{e^{\mu_{max}(t-t_0)} + 1} \tag{20}$$

As we have colony area data, we can fit the model to them and compute $\mu(t)$ value. Also with the area values we can compute I/A directly from the data and smooth them with a spline (using scipy univariate spline) to compute the derivative of that function. Then we are able to compute the $K_F(t)$ value on (15).
