# Supplementary material for: Low cost and open source multi-fluorescence imaging system for teaching and research in biology and bioengineering
Source: PLoS One. 2017 Nov 15;12(11):e0187163. doi: 10.1371/journal.pone.0187163 (PMC5687719; doi:10.1371/journal.pone.0187163)
Supplement: S6 File — (PDF) [file pone.0187163.s008.pdf]

Colony classificator maths

September 29, 2017

0.1 Colony classificator maths

Through approximating the relation between the fluorescent protein level with the fluorescence intensity as a linear one, it's possible to write:

$$\text{Fluorescence intensity vector} = A \cdot \text{fluorescence protein level vector} + \text{basal signal}$$

$$\begin{bmatrix} R(t) \\ G(t) \\ B(t) \end{bmatrix} = \begin{bmatrix} a_{RR} & a_{GR} & a_{OR} \\ a_{RG} & a_{GG} & a_{OG} \\ a_{RB} & a_{GB} & a_{OB} \end{bmatrix} \begin{bmatrix} FP_R \\ FP_G \\ FP_O \end{bmatrix} + \begin{bmatrix} R_0 \\ G_0 \\ B_0 \end{bmatrix}$$

where A is the linearization matrix that contains the liner relation slopes values, which correspond to the amount of signal produced by each unit of fluorescent protein concentration $\left(e.g.a_{RR} = \text{red signal produced per unit of } FP_R, \, a_{RG} = \text{green signal produced per unit of } FP_R, etc\right)$

If it have only one fluorescent protein per colony, let's say $FP_R > 0$, $FP_G = 0$, $FP_O = 0$, then:

$$\begin{bmatrix} R(t) \\ G(t) \\ B(t) \end{bmatrix} = \begin{bmatrix} a_{RR} & a_{GR} & a_{OR} \\ a_{RG} & a_{GG} & a_{OG} \\ a_{RB} & a_{GB} & a_{OB} \end{bmatrix} \begin{bmatrix} FP_R \\ 0 \\ 0 \end{bmatrix} + \begin{bmatrix} R_0 \\ G_0 \\ B_0 \end{bmatrix}$$

That is equal to:

$$\begin{bmatrix} R(t) \\ G(t) \\ B(t) \end{bmatrix} = \begin{bmatrix} FP_R \cdot a_{RR} \\ FP_R \cdot a_{RG} \\ FP_R \cdot a_{RB} \end{bmatrix} + \begin{bmatrix} R_0 \\ G_0 \\ B_0 \end{bmatrix}$$

By taking only the red and green channels we can obtain a 2 eq. system:

$$\begin{array}{ll} (1) & R(t) = FP_R \cdot a_{RR} + R_0 \\ (2) & G(t) = FP_R \cdot a_{RG} + G_0 \end{array}$$

Assuming $a_{RG} \neq 0$, rearrange (2) as:

$$FP_R = \frac{G(t) - G_0}{a_{RG}}$$

and replacing $FP_R $ on (1):

$$R(t) = \frac{G(t) - G_0}{a_{RG}} \cdot a_{RR} + R_0$$

Rearranging it gives:

$$R(t) = G(t)\frac{a_{RR}}{a_{RG}} - \frac{G_0 \cdot a_{RR}}{a_{RG}} + R_0$$

Finally, condensing the constants it becomes a linear relation between $R(t)$ and $G(t)$.:

$$R(t) = G(t) \cdot m_R + b_R$$

where $m_R = \frac{a_{RR}}{a_{RG}}$ and $b_R = R_0 - \frac{G_0 * a_{RR}}{a_{RG}}$, which are intrinsic parameters of each fluorescent protein.

By proceeding the same way, it is possible to obtain a characteristic linear expression for each fluorescent protein:

when $FP_R = 0$, $FP_G > 0$, $FP_O = 0$:

$$R(t) = G(t) \cdot m_G + b_G$$

when $FP_R = 0$, $FP_G = 0$, $FP_O > 0$:

$$R(t) = G(t) \cdot m_O + b_O$$
